# Do They Know What They Are Doing? Cognitive Aspects of Rescue Behaviour Directed by Workers of the Red Wood Ant *Formica polyctena* to Nestmate Victims Entrapped in Artificial Snares

**DOI:** 10.3390/life14040515

**Published:** 2024-04-16

**Authors:** Anna Szczuka, Alicja Sochacka-Marlowe, Julita Korczyńska, Paweł Jarosław Mazurkiewicz, Beata Symonowicz, Olga Kukina, Ewa Joanna Godzińska

**Affiliations:** 1 Laboratory of Ethology, Nencki Institute of Experimental Biology of the Polish Academy of Sciences, Ludwika Pasteura St. 3, PL 02-093 Warsaw, Poland; a.szczuka@nencki.edu.pl (A.S.); alamarlowe@gmail.com (A.S.-M.); j.korczynska@nencki.edu.pl (J.K.); pawel.j.mazurkiewicz@gmail.com (P.J.M.); beata_sym@o2.pl (B.S.); ol.kukina@gmail.com (O.K.); 2 Department of Biology and Integrated Bioscience Program, University of Akron, Akron, OH 44325, USA; 3 College of Inter-Faculty Individual Studies in Mathematics and Natural Sciences (MISMaP), University of Warsaw, Stefana Banacha St. 2c, PL 02-097 Warsaw, Poland; 4 Department of Entomology, Phytopathology and Physiology, Ukrainian Research Institute of Forestry and Forest Melioration, Pushkinska St. 86, 61024 Kharkiv, Ukraine

**Keywords:** altruism, prosocial behaviour, rescue behaviour, cognitive processes, social insects, Hymenoptera, Formicidae, *Formica polyctena*

## Abstract

Ant rescue behaviour belongs to the most interesting subcategories of prosocial and altruistic behaviour encountered in the animal world. Several studies suggested that ants are able to identify what exactly restrains the movements of another individual and to direct their rescue behaviour precisely to that object. To shed more light on the question of how precise the identification of the source of restraint of another ant is, we investigated rescue behaviour of red wood ant *Formica polyctena* workers, using a new version of an artificial snare bioassay in which a nestmate victim bore two wire loops on its body, one (acting as a snare) placed on its petiole and an additional one on its leg. The tested ants did not preferentially direct their rescue behaviour towards the snare. Moreover, the overall strategy adopted by the most active rescuers was not limited to precisely targeted rescue attempts directed towards the snare, but consisted of frequent switching between various subcategories of rescue behaviour. These findings highlight the importance of precise identification of cognitive processes and overall behavioural strategies for better understanding of causal factors underlying animal helping behaviour in light of new facts discovered by testing of various successive research hypotheses.

## 1. Introduction

### 1.1. Affiliative Behaviour, Prosocial Behaviour, Cooperation, Altruism, Helping Behaviour

Behavioural correlates of a social mode of life include affiliative behaviour, a wide spectrum of non-aggressive (friendly) social contacts and interactions [1,2]. Theoretical and experimental research devoted to that issue involves the use of numerous synonymous notions such as prosocial behaviour (pro-sociality) [3,4,5,6,7,8,9,10,11,12,13,14], cooperative behaviour (cooperation) [11,15,16,17,18,19,20], altruistic behaviour (altruism) [11,16,17,19,20,21,22,23,24], helping behaviour [3,4,5,16,17,18,25,26] and rescue behaviour [18,19,20,26,27,28,29,30]. The exact scopes of these notions are not easy to delineate, as they are not always defined in precisely the same way. The most general of these terms, prosocial behaviour, is most frequently used to denote activities that bring benefits to other individuals [3,4,6,7,8,9,10,11,12,13,14]. The notions of cooperation and altruism are more narrow. Whereas cooperative behaviour brings benefits to both actors and recipients of such acts [16,17,20], altruistic behaviour involves actions that bring benefits to other individuals but are associated with costs for the altruist, or at least with serious risks associated with such costs [11,16,17,19,20,21,22,23,24]. Lastly, helping behaviour is associated with benefits received either solely by its recipient, or by both the recipient and the actor and, therefore, encompasses both cooperation and altruism [16,17]. Helping behaviour is also sometimes identified with prosocial behaviour [17], although according to some authors, prosocial behaviour includes other subcategories besides helping [12]. The term “helping behaviour” is also sometimes used in an interchangeable way with the term “altruistic behaviour” [22] and relatively frequently has also been used to label rescue behaviour [3,4,5,12,31,32,33,34,35,36,37,38,39].

### 1.2. Rescue Behaviour: Definition and Criteria

Rescue behaviour, one of the most interesting subcategories of risky pro-social behaviour, is usually defined as a social interaction during which one individual, the victim, is endangered and another individual, the rescuer, places itself at risk of costs and fitness losses by engaging in rescue attempts. The behaviour of the rescuer should also be suited to the circumstances and should lead to the decrease or elimination of the victim’s distress and/or danger. Lastly, rescue behaviour should not be inherently rewarding or beneficial to the rescuer, although it may be followed by indirect advantages [18,19,20].

We also should bear in mind that not all behaviour directed towards an endangered victim belongs to the category of rescue behaviour. For instance contacting the victim, but without providing to it any help, should not be classified as rescue behaviour [20]. Sometimes it is also difficult to tell apart rescue behaviour from cooperative self-defence [18,40,41].

Various forms of rescue behaviour have been extensively documented in both vertebrates and invertebrates in a wide range of naturally occurring and experimentally created contexts and situations (for the reviews, see [7,18,19,20,41,42,43,44]). Rescue behaviour is extremely interesting for the students of behaviour, not only due to its causation and evolution, but also because the research devoted to various aspects of rescue activities not only throws light on proximate and ultimate causal factors underlying risky altruistic behaviour, but also contributes to broadening of our knowledge about inter-specific variability of prosocial behaviour and about cognitive processes intervening in its mediation in various animal groups.

### 1.3. Ant Rescue Behaviour: Contexts and Bioassays

Rescue behaviour displayed by the ants in response to endangered nestmates belongs to the most interesting manifestations of prosocial behaviour encountered in the animal world. Ant rescue behaviour was first described by the British naturalist Thomas Belt (1874) during his field research in Nicaragua [45]. When observing a moving column of *Eciton hamatum* army ants, Belt immobilized one of the ants by placing a small stone on its body. The nestmates of the victim rushed to its rescue. Some of them bit at the stone, others seized the trapped ant by the legs and tugged at them until they freed the victim. Several years later, another British naturalist John Lubbock (1882) [46] reported that crippled workers of *Formica fusca* and workers of *Lasius flavus* inebriated with ethanol were taken by their nestmates to the nest where crippled individuals could live safely and inebriated ones could recover. Interestingly, many years later, in the years 2017 and 2018, similar rescue behaviour was also documented in workers of the termite-hunting ponerine ant species *Megaponera analis*. Ants of that species were observed to transport injured nestmates back to the nest and to engage in intense allogrooming that facilitated their wound healing [47,48].

Further reports documenting the occurrence of rescue behaviour in ants from various species and subfamilies were focused mostly on the responses of these insects to nestmates immobilized by inanimate obstacles: buried in soil, sand or clay [49,50,51,52,53,54,55] or imprisoned in a container closed by a stopper made partly of cardboard [56]. Ant rescue activities reported in these studies included digging close to the buried individual, biting the cardboard and pulling at the victim’s extremities. However, these early studies were focused mostly on the identification of chemical and vibrational stimuli eliciting alarm and digging and biting behaviour rather than on the importance of these behaviour patterns as manifestations of prosocial behaviour [51,52,53,54,55,56,57,58].

About twenty years ago, Czechowski et al. (2002) [28] employed the term “rescue behaviour” in the title and the text of the paper describing rescue behaviour displayed by workers of three ant species (*Formica sanguinea*, *Formica fusca* and *Formica cinerea*) in response to ant victims captured by predatory antlion larvae (*Myrmeleon formicarius*). This study provided inspiration for further extensive research carried out both in the field and in the laboratory with the use of two main bioassays: antlion larva capture bioassay, during which ant rescue behaviour was elicited in response to an ant captured by an antlion larva [59,60,61,62,63] and artificial snare bioassay, during which ant rescue behaviour was elicited in response to a victim ant entrapped in an artificial snare [29,59,61,62,64,65,66,67,68,69,70,71,72,73,74,75,76,77].

The classical version of the artificial snare bioassay was introduced by Nowbahari et al. (2009) [29] in a study carried out to investigate rescue behaviour of workers of sand-dwelling formicine ant species *Cataglyphis piliscapa* (then known as *Cataglyphis cursor*). The victim ant was fixed to a small piece of filter paper by means of a thin nylon loop passing over its petiole (the narrow part of the body between the thorax and the gaster). The snare with the victim was then partly buried in dry sand. The use of the filter paper as a part of an artificial snare apparatus was expected to help to trap volatile pheromones emitted by the victim. Actually, rescuer ants were observed to approach and contact pieces of filter paper soiled by previous victims that were no longer fixed to them [30].

Such an artificial snare bioassay has been used in numerous laboratory and field experiments to test a wide range of ant species from the subfamilies Formicinae [29,59,61,62,64,65,66,67,68,69,70,72,74,75,76,77], Myrmicinae [59,61,67] and Dolichoderinae [61]. Modified versions of that bioassay consisted of a confrontation of potential rescuers with a victim immobilized by means of duct tape applied to its legs (*Odontomachus brunneus*, subfamily Ponerinae) [73] and of simultaneous confrontation of potential rescuers with two artificial snares, one empty and one containing a victim, or containing two different victims (*Cataglyphis nigra*, subfamily Formicinae) [77]. Yet more studies of ant rescue behaviour investigated the responses of weaver ants *Oecophylla smaragdina* (subfamily Formicinae) and harvester ants *Veromessor pergandei* (subfamily Myrmicinae) to victims wrapped in spider silk [71,78]. A probable case of rescue of a nestmate from the web of an agelenid funnel spider was also reported in *Formica pratensis* (Kupryjanowicz and Włodarczyk, unpublished observation reported in [41,79,80]).

### 1.4. Cognitive Abilities of Social Insects

A rapidly growing body of results from numerous experimental and theoretical studies have brought about increasingly convincing evidence that the cognitive processes of social insects may be highly advanced and sophisticated [81,82,83]. To give just a few examples, for a long time, it has been known that honeybee foragers use symbolic communication (dance language) to provide to their nestmates precise information about encountered food sources [84,85]. *Polistes fuscatus* social wasps can learn to individually identify their nestmates on the basis of their unique facial coloration patterns [86,87] and can assess fighting ability of potential rivals solely on the basis of their observation, without risky direct contact (so-called social eavesdropping) [88]. Similar observational learning was also documented in bumblebees, which proved to be able to learn from trained demonstrators how to manipulate specific objects in order to gain food rewards [89,90].

The cognitive abilities of ants are no less spectacular [81,82,83] and include, among others, cooperative transport of objects too large to be moved by a single individual [91,92,93], individual recognition of specific nestmates on the basis of chemical cues present on their body surface [94], tool use [95,96,97], ability to count steps to evaluate distances [98], rapid learning to avoid antlion traps following a single successful escape from a pit [99] and teaching of naive individuals by the more experienced ones [100,101].

### 1.5. Cognitive Aspects of Ant Rescue Behaviour

Two recent reviews on insect cognition, the first one devoted to advanced cognition in ants [82] and the second one focusing on social cognition of both eusocial and non-eusocial insects [83], have provided exhaustive reviews of a wide range of issues, but, surprisingly, they did not discuss or even mention ant rescue behaviour. Such omissions should be avoided in future reviews devoted to advanced cognition in both invertebrates in general and ants in particular, as they generate an incomplete image of the current state of the art in both these domains of research.

However, in another recent review [81], ant rescue behaviour is presented as one of the most interesting examples of cognitive abilities of social insects. Nevertheless, the question of the precision with which the ants identify the sources of the problems of victims they try to rescue is not discussed in that paper. Yet that particular question belongs to the most important issues raised by the research on ant rescue behaviour.

The findings of many studies carried out to shed more light on causal factors involved in the mediation of ant rescue behaviour strongly suggest that ant rescuers are able to identify precisely what restrains the movements of another individual and to direct their rescue attempts toward that specific obstacle. Such an ability was documented mostly in the results of the experiments carried out with the use of the artificial snare bioassay, including the first study in which this method has been introduced [29]. In these studies, rescue attempts directed precisely to the object responsible for the victim’s entrapment were labelled with several similar terms, including such expressions as “precision rescue”, “precision rescue behaviour” and “precise rescue behaviour patterns” [19,59,64,65,67,74,102]; “precisely directed rescue behaviour” [29,30,68,102]; “precisely targeted rescue behaviour” [30,66,68,72]; and “precisely tuned rescue behaviour” [80]. Responses to the snare responsible for the entrapment of the victim were also investigated and/or discussed in some other research papers in which these terms were not used [61,62,70,73,75,76], as well as in several review papers [11,19,41,80]. However, surprisingly, in some studies, pulling at the snare and at the victim’s appendages were not analysed separately, but pooled together to form a more general subcategory of pulling behaviour [68,77].

Precise targeting of the source of the victim’s entrapment was also observed in the context of rescue of victims of antlion larvae. Several authors highlighted many analogies between behaviour patterns directed by ant rescuers towards artificial snares and towards antlions holding the victims in their mandibles [59,68,72,80,102]. In both cases rescue attempts included biting and pulling behaviour directed either to the snare, or to the antlion. Attacking of an antlion larva that had captured a conspecific ant was reported in the formicine ants *Formica sanguinea* [28] and *Formica cinerea* [28,60,61,62,63] and in the myrmecine ants *Tetramorium* sp. E [59]. Ants from that species were also observed to sting the antlion that had captured their nestmate [59]. Interestingly, stinging behaviour was also observed in the ponerine ants *Odontomachus brunneus* tested by means of a modified variant of artificial snare bioassay. The rescuers directed it to the duct tape used to immobilize the legs of conspecific victims [73].

It should, however, be remembered that although all these findings strongly suggest that precisely targeted rescue behaviour documented by them in the ants is controlled by advanced cognitive processes, the involvement of such processes in the mediation of that behaviour has not yet been proved in a fully unequivocal way.

Another important subcategory of causal factors involved in the mediation of ant rescue behaviour includes cognitive processes underlying successive choices of specific subcategories of rescue attempts. In particular, detailed analysis of behavioural sequences performed by workers of *Cataglyphis piliscapa* in response to nestmate victims entrapped in artificial snares revealed that these sequences did not consist either of fixed behaviour patterns, or of series of random acts, and that successive decisions of rescuers were significantly influenced by memories of their past actions [68]. Similarly, workers of another species from the genus *Cataglyphis*, *Cataglyphis nigra*, were found to adapt their rescue behaviour to the specific requirements of different victims entrapped in artificial snares [77]. 

Lastly, numerous studies analysed causal factors underlying ant rescue behaviour in terms of the evolution of that behaviour and/or its dependence on the ecology of the tested ant species [11,19,20,28,30,41,59,61,67,80].

### 1.6. The Aim of the Present Study

The main aim of the present study was to shed more light on cognitive processes involved in the mediation of ant rescue behaviour and, in particular, on the ability of the ants to identify what exactly restrains the movements of another individual and, as a consequence, to engage in precisely targeted rescue attempts. To that purpose, in the present study, we used a new, modified version of the artificial snare bioassay. Similarly to many previous experiments, potential ant rescuers were confronted with a nestmate victim fixed to a filter paper disc by a thin wire loop drawn over the petiole. However, this time, a second, closely similar wire loop was placed on the victim’s leg. Whereas the wire loop on the victim’s petiole acted as a snare, the loop on the leg did not play any role in the victim’s entrapment. We assumed that precise identification of the object responsible for the victim’s immobilization should be followed by precisely targeted rescue behaviour directed towards that object. However, if rescue behaviour of the tested ants were directed towards both wire loops with no preference for the loop on the victim’s petiole acting as a snare, such a finding would strongly suggest that the responses of the rescuer ants to the wire loops placed on the victim’s body consisted of attempts to remove foreign objects adhering to the victim’s body surface rather than of precisely targeted responses to the source of the victim’s entrapment.

Additionally, we also expected that our experiment would broaden our knowledge about the diversity, variability and individual differences in rescue activities carried out by the nestmates from the same ant colony in a specific experimental situation.

**Figure 1:**
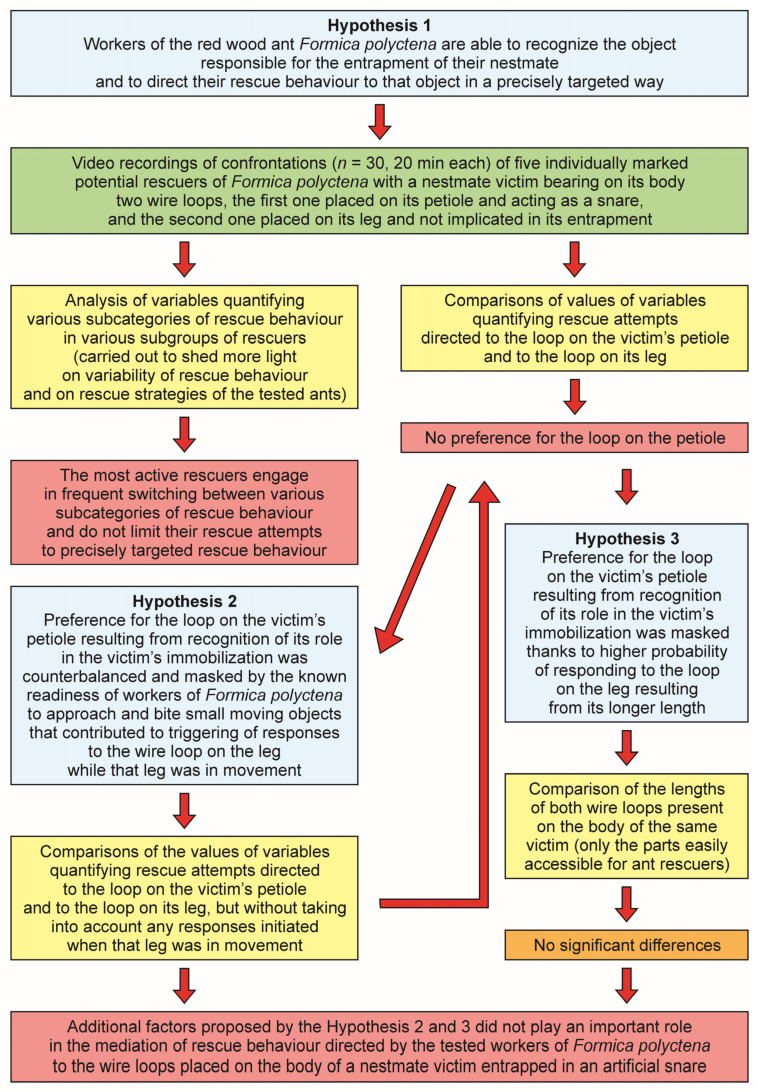
A diagram showing the main elements of the experimental design used in the present study (hypotheses, methods, results and conclusions).

The main elements of the experimental design used in the present study (hypotheses, methods, results and conclusions) are shown in form of a diagram in Figure 1.

### 1.7. Ants Used as Subjects in This Study: Species and Worker Status

We used as subjects foragers of the red wood ant species *Formica polyctena* (Hymenoptera: Formicidae, subfamily Formicinae, group *Formica rufa*). These mound-building ants are fairly common in Poland and in the large part of both the European and the Asian Palearctic [103]. They often form huge polydomous (polycalic) colonies composed of complex systems of numerous interconnected nests [103] and are known to play an important role in forest protection [104,105,106]. 

Causal factors involved in the mediation of behaviour of *Formica polyctena* workers have already been investigated in numerous studies exploring such issues as neurochemical and social determinants of their aggressive and predatory behaviour [107,108,109,110,111,112,113,114], affiliative and prosocial behaviour (including interactions with adult nestmates and with brood) [61,113,115,116] and responses to various elements of physical environment [117,118,119,120] (see also [121]). In particular, propensity to engage in rescue behaviour has already been documented in workers of that species in a study with the use of two types of bioassays, antlion larva capture bioassay and artificial snare bioassay [61].

In ant societies, workers as a rule engage first in intranidal (inside-nest) activities and then switch to extranidal (outside-nest) tasks as they age (the so called nurse–forager transition) [91,122,123,124,125]. In this study, only extranidal workers, foragers, were used as both potential rescuers and as victims. As shown by the studies investigating the importance of age and/or behavioural specialization on the expression of rescue behaviour in workers of another formicine ant species, *Cataglyphis piliscapa*, foragers are the most active as rescuers [64,65] and are also able to obtain the most help when acting as victims [64].

## 2. Materials and Methods 

### 2.1. Ants

Workers of the red wood ant *Formica polyctena* used in this experiment were collected from an ant hill located in a mixed pine forest near Krześlin (central–eastern Poland) (GPS coordinates: 52.242161 N, 22.376328 E) on 2 July 2010.

The collected colony fragment (about 7 thousand workers) was transferred to the Laboratory of Ethology of the Nencki Institute of Experimental Biology of the Polish Academy of Sciences in Warsaw and housed in an artificial nest composed of four rectangular Perspex containers (30 cm × 20 cm, 15 cm high) connected by small (5.5 cm long, inner diameter 1.5 cm) pieces of silicone tubing. The bottom of each container was covered with a thin (about 1 cm) layer of fine dry sand, and the inner surface of its walls was coated with Fluon^®^ (PTFE), a substance providing a silky smooth surface and commonly used in myrmecological research to prevent ants from escaping from artificial nests. Two of these boxes each contained about a dozen large (200 mm long, inner diameter 20 mm) glass test tubes acting as artificial nest chambers. Each tube contained an about 2 cm long reservoir of water trapped in by means of a tightly fitting cotton plug to create a humidity gradient allowing the ants to choose their preferred humidity conditions. The tubes were shielded from light by a sheet of aluminium foil. The remaining two boxes served as foraging areas. Food and water were provided on small (5 cm in diameter) Petri dishes. Food consisted of honey mixed with crushed apples and sand (added to make the mixture less sticky) and of pieces of house crickets (*Acheta domesticus*) killed by freezing and then allowed to thaw at room temperature. Carbohydrate and proteinic food was placed on two separate Petri dishes and exchanged for a fresh one three times a week. Drinking water was provided on similar small Petri dishes filled with moist cotton. The nests were kept at a fairly stable ambient temperature (25 ± 1 °C) and relative humidity of the air (30–39%) and exposed to a natural rhythm of daylight and darkness supplemented with artificial white light illumination provided by a FOTOVITA FV 10 L daylight lamp and delivered at 12:12 LD.

### 2.2. Tests

#### 2.2.1. Preliminary Preparations

All ants used as subjects in the nestmate rescue tests, both as potential rescuers and as victims, were taken from among foragers present in the foraging areas of the main nest. All manipulations were made by persons wearing disposable gloves and using fine entomological tweezers.

Ants intended to be used as potential rescuers were individually marked with the use of waterproof Edding markers at least one day before the test in which they had to participate. A single dot of quick-drying paint (red, blue, green, yellow or violet) was placed on the thorax of the marked ant worker while it was gently held by the experimenter. Freshly marked ants were then placed in a small cylindrical glass crystallizer (10 cm in diameter, 5 cm high) with the walls covered with Fluon^®^ and observed during a few minutes to make sure that they had not been damaged during the marking process or the subsequent self-grooming. They were returned again to the foraging area of the main nest when they were already entirely dry.

Foragers of *Formica polyctena* intended to be used as victims were not marked, as only a single victim was used during each test.

The tests (*n* = 30, 20 min each) were performed on five successive days (11–15 December 2010). Dry sand used to cover the bottom of the containers in which the potential rescuers were confronted with victims was first marked with odour cues by the nestmates of the tested ants. Sand marking was always carried out about one hour before the start of the first test performed on the given day. The ants that had to be used to mark the sand (*n* = 100) were captured with the help of tweezers in the foraging area of the main nest on the first test day and were then used to mark the sand also on the remaining four test days. 

Sand marking was carried out by placing a 1.5 cm layer of clean dry sand in a cylindrical glass container (23 cm in diameter, 9.5 cm high) with the walls covered with Fluon^®^ and then introducing 100 freely moving foragers into that container. After one hour, these ants were gently removed from the container with sand and placed in an artificial nest made of a single rectangular Perspex container (30 cm × 20 cm × 15 cm) with the walls covered with Fluon^®^. That nest contained a hiding place made of a large glass test tube equipped with a water reservoir and shielded from light by a bent piece of aluminium foil. Carbohydrate food (the same as in the case of the main ant nest) and moist cotton serving as the source of drinking water were offered on two small (3 cm in diameter) Petri dishes. After the end of the experiment, the ants used to mark the sand were returned again to the main nest.

#### 2.2.2. Nestmate Rescue Tests

**Figure 2:**
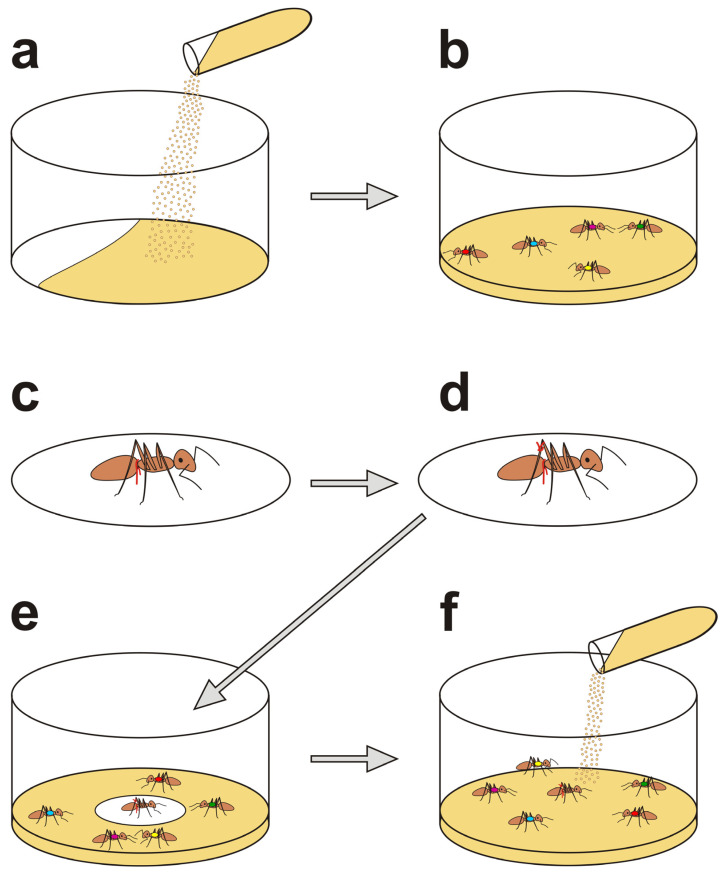
Main successive stages of the preparation of an artificial snare bioassay with two wire loops placed on the body of the entrapped victim ant. (**a**) Preparing the experimental arena: placing dry sand marked with chemical cues left by nestmates of the tested ants in a small crystallizer. (**b**) Placing five foragers of *Formica polyctena* (marked individually with paint) in the experimental arena and allowing them to habituate for 10 min. (**c**) Placing a forager from the same ant colony in an artificial snare (tying it to a circular piece of filter paper by means of a thin wire loop passing over its petiole). (**d**) Placing the second wire loop on the tibia of the victim’s right hind leg. (**e**) Placing the victim entrapped in an artificial snare in the experimental arena. (**f**) Sprinkling the central part of the arena with marked dry sand to bury the paper part of the snare apparatus.

The main successive stages of the preparation of our novel version of an artificial snare bioassay with two wire loops placed on the body of the entrapped victim ant are shown in Figure 2.

Nestmate rescue tests were carried out in experimental arenas made of cylindrical glass containers (9.5 cm in diameter, 5 cm high) with the inner surface of the walls covered with Fluon^®^. The floor of each arena was covered with a 0.5 cm layer of dry sand that had been marked with odour cues by the nestmates of the tested ants earlier on the same day.

Before the start of each test, five *Formica polyctena* foragers that at least one day earlier had been marked individually with different colours were captured in the foraging area of the main ant nest, placed in the test arena and left there for 10 min to undergo habituation to experimental conditions. During that 10 min period, another ant from the same colony (an unmarked one) was captured in the foraging area of the main nest and fixed to a circular filter paper disc (1.5 cm in diameter) by means of a loop made of thin copper wire (0.1 mm in diameter) drawn through two small holes in the paper (about 2 mm apart). The ant was placed gently within the loop by means of tweezers, and the wire was then pulled downwards to hold the victim down by passing over its petiole. The free ends of the wire were then tied below the underside of the disc. An additional loop of the same wire was then tied on the tibia of the right hind leg of the victim, and the loose ends of the wire were cut off with sharp scissors. That wire loop did not play any role in tying the victim to the paper part of the snare apparatus. The length of the wire accessible for the potential rescuers on the victim’s leg was determined by measuring (with the use of callipers) the whole piece of wire before placing it on the ant’s leg and then measuring the pieces that have been cut off. The length of the part of the other wire loop accessible for the rescuers on the victim’s petiole was determined after the test (see below).

At the end of the 10 min period of habituation of potential rescuers to the experimental arena, the disc with the attached victim was placed in the centre of the arena and sprinkled with marked dry sand, leaving exposed only the immobilized victim. Only the ants that did not eject formic acid while they were being tied to the paper disc or immediately afterwards were used in the experiment. Ejection of formic acid can be easily perceived by the experimenters as it is accompanied by the detectable smell of that compound.

The tested ants were exposed to artificial white light illumination produced by the daylight lamp FOTOVITA FV-10 L placed 40 cm from the arena. The illumination level measured at the centre of the arena (3800 lx) was lower than the usual illumination level provided by that lamp at that distance, as the light attaining the centre of the arena had to pass through its side walls covered by a non-transparent white layer of Fluon^®^. Additional, much less strong sources of light present in the laboratory room included incandescent white light produced by ordinary lamps placed close to its ceiling and sunlight (without the UV component) arriving through the window glass.

The tests were recorded by means of the digital video camcorder Canon XL2 mounted on a tripod to allow top view recording. To facilitate precise identification of specific subcategories of rescue attempts, only behaviour patterns taking place in the central part of each experimental arena (4 cm × 5.5 cm) were recorded. For each test we used a fresh arena and fresh sand.

After the test the victim ant was freed from the snare by cutting the wire loop on its petiole in two places, as closely as possible to the upper surface of the paper. The part of the wire loop that during the test was fully exposed for the rescuers was then measured with the help of callipers. The part of the wire that was hidden under the paper disc was not taken into account in the comparisons of the length of the wire loops placed on the victim’s leg and on its petiole, as the rescuer ants had very limited access to it. During the whole experiment crawling under the paper was observed very infrequently (only in the case of 6 out of 150 tested ants) and it was impossible to tell if the ant responded or did not respond to the part of the wire placed under the paper, as it was hidden from the view of the camcorder.

### 2.3. Analysis of Behavioural Recordings: Behavioural Categories Quantifying Rescue Attempts

Behaviour displayed by *Formica polyctena* workers during nestmate rescue tests was analysed by means of the software “The Observer Video-Pro” (Noldus Information Technology) [126]. We quantified the behaviour of the tested ants, taking into account 36 behavioural categories. Out of that number, 15 behavioural categories quantified rescue attempts of individually marked freely moving ants (see Table 1).

Examples of various subcategories of rescue behaviour recorded during our tests can also be seen in five short (43″–1′59″) videos (S1–S5) placed by us in the Supplementary Online Materials together with a PDF File S1 with the explanations of their contents.

The results of the analysis of the variables quantifying the remaining 21 behavioural categories used by us during the analysis of our recordings will be reported in a separate publication.

### 2.4. Statistical Analysis of the Data

#### 2.4.1. Variables Calculated to Quantify Ant Rescue Behaviour

The rate of occurrence of rescue behaviour observed during our experiment was analysed in two ways as the rate of occurrence of the tests during which that behaviour was observed (the ratio of the tests during which the behaviour in question was present to those during which it was absent) and as the share of the ants that engaged in that behaviour in the total number of the tested workers [*n* = 150 (30 tests × 5 workers)]. These rescuer ants (*n* = 53) were then further divided into two subgroups: ants that engaged in rescue attempts, but never directed their behaviour to any of the wire loops (WL) placed on the body of the victim (WL− ants, *n* = 17) and ants that were observed to respond to one or both of these wire loops (WL) (WL+ ants, *n* = 36). WL+ ants were then subdivided into three further subgroups: ants that responded only to the wire loop placed on the victim’s leg (L ants, *n* = 12), ants that responded only to the wire loop placed on the victim’s petiole (P ants, *n* = 12) and ants that responded to both loops (L+P ants, *n* = 12).

For the purpose of further analysis, categories used to quantify ant rescue behaviour during the analysis of behavioural recordings were pooled into the following more general categories:

(1) Responses to the victim’s body
(2) Responses to the substrate near the victim
(3) Rescue behaviour not involving responses to the wire loops placed on the victim’s body (1 + 2)
(4) Responses to the wire loop on the victim’s petiole
(5) Responses to the wire loop on the victim’s leg
(6) Responses to any of the wire loops placed on the victim’s body (4 + 5)
(7) All subcategories of rescue behaviour taken together (1 + 2 + 4 + 5).

Each of these behavioural categories was quantified by three variables:

- the latency from the start of the test to the first episode of the behaviour in question (expressed as the percent of the total test time to make possible taking into account also the ants that did not display that behaviour)
- the number of episodes of that behaviour recorded during test
- the total duration of all episodes of that behaviour recorded during the test.

It should be added that the numbers of episodes of these main subcategories of rescue behaviour were not calculated by automatically adding the numbers of episodes of more precisely defined behavioural subcategories. If activities belonging to the same main subcategory of rescue behaviour were carried out in succession and were not separated by activities not belonging to that behavioural subcategory, they were pooled and considered to represent a single episode of that particular behaviour.

We also analysed sequences of successive subcategories of rescue behaviour displayed by individual ants. In this analysis, successive bouts of the same subcategory of rescue behaviour were treated as a single element of the sequence if they were not separated by behaviour belonging to another subcategory of rescue attempts, even if they were interspersed with bouts of behaviour not consisting of rescue attempts.

The rates of occurrence of various subcategories of rescue behaviour were also calculated and analysed.

The main question asked by the present study (do the ants prefer to direct their rescue behaviour towards the wire loop responsible for the immobilization of the victim?) was addressed by comparing the values of the variables calculated to quantify the responses of the tested WL+ ants to the loop on the victim’s leg (not implicated in its immobilization) and to the loop on its petiole (responsible for its immobilization).

We also considered a possibility that the responses of *Formica polyctena* workers to the wire loop on the leg of the victim might have been at least partly influenced by the known propensity of these ants to be attracted to small moving objects and to respond to them with biting behaviour [107,108,109,110,111,112,113,114,115,116,117]. To eliminate the possible influence of that factor, we additionally compared the responses of WL+ ants to two wire loops placed on the body of the victim after having discarded from the analysis all responses to the wire loop on the victim’s leg that were initiated while that leg was in movement.

#### 2.4.2. Statistical Tests Used in the Analysis of the Data

The data obtained in this experiment were analysed with the use of non-parametric tests (software: SPSS IBM Statistics version 25). At the start of the analysis, rescue behaviour of each rescuer (*n* = 53) was divided into three main subcategories: rescue attempts directed towards the victim’s body, to the substrate near the victim and to the wire loops placed on the victim’s body. To find out which subcategory of rescue behaviour was predominant and which one was the least well expressed during our experiment, these data were then analysed by means of Friedman ANOVA followed by Dunn–Sidak post hoc tests for dependent data. We carried out three separate analyses, one for each of the three variables used to characterize the behaviour of the rescuers (the latency from the start of the test to the first episode of the analysed behaviour, the number of episodes of that behaviour recorded during the test and the total duration of all episodes of that behaviour).

The same three variables (latency, number of episodes and total duration) were also calculated for two further subcategories of rescue behaviour: all subcategories of rescue behaviour taken together and rescue behaviour not involving responses to the wire loops placed on the body of the victim. The values of these variables were calculated separately for two subgroups of rescuers, WL− ants (*n* = 17) and WL+ ones (*n* = 36) and then compared by means of the two-tailed Mann–Whitney U test.

Rescue behaviour in general (all subcategories pooled together) was also quantified by these three variables calculated separately for L, L+P and P ants (*n* = 12 in each subgroup). These three analyses were carried out with the use of the Kruskal–Wallis ANOVA followed by Dunn–Sidak post hoc tests for independent data. Kruskal–Wallis ANOVA followed by Dunn–Sidak post hoc tests for independent data was also used to compare total durations of responses to the wire loops carried out by L, L+P and P ants.

The numbers of the elements of the sequences of successive subcategories of rescue behaviour were compared by means of the two-tailed Mann–Whitney U test (in the case of the comparison of WL− and WL+ ants) and by means of the Kruskal–Wallis ANOVA followed by Dunn–Sidak post hoc tests for independent data (in the case of the comparison of L, L+P and P ants).

The rates of occurrence of specific subcategories of rescue behaviour in two subgroups of rescuers, WL− ants and WL+ ones, were calculated in two ways, taking into account all responses of the tested ants and taking into account solely the first episodes of rescue behaviour recorded during the test. The data obtained for WL− ants and WL+ ones were then compared by means of the two-tailed Fisher Exact Probability Test.

The comparisons of the values of three variables quantifying the responses of WL+ ants to the wire loop placed on the victim’s leg (L) and on the victim’s petiole (P) (latency, number of episodes and their total duration) were carried out with the use of the Wilcoxon matched-pairs signed-rank test. Altogether, six such tests were performed, three taking into account all obtained data and three carried out without taking into account the responses to the wire loop on the victim’s leg taking place when that leg was in movement.

Lastly, the lengths of the wire loops placed on the leg (L) and the petiole (P) of each victim (*n* = 30) were compared with the use of Wilcoxon matched-pairs signed-rank test.

## 3. Results

### 3.1. Occurrence of Rescue Behaviour during Artificial Snare Bioassays

Workers of the red wood ant *Formica polyctena* engaged in rescue behaviour during the majority of the confrontations with a nestmate victim entrapped in an artificial snare (25 out of 30 tests; 83.3%). Responses to one or both of the wire loops placed on the body of the victim were also recorded on the majority of the tests (22 out of 30; 73.3%). During three tests (10% of the total number of tests) the ants engaged in rescue attempts, but did not direct them to any of the wire loops placed on the victim’s body.

**Figure 3:**
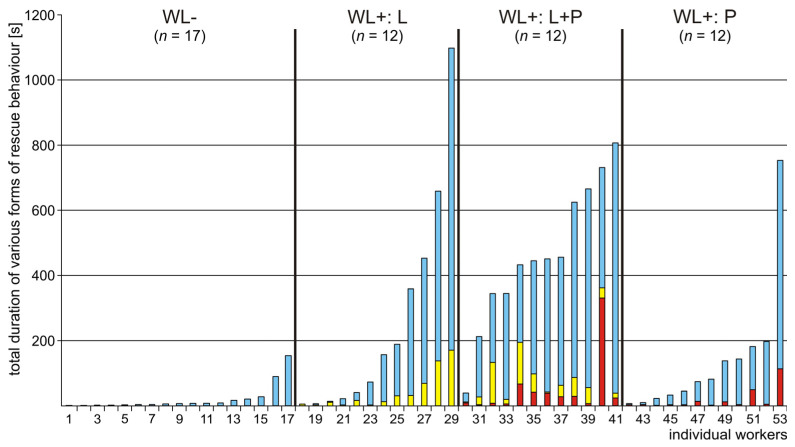
Total duration of various subcategories of rescue behaviour displayed by individual workers of the red wood ant *Formica polyctena* (*n* = 53) in response to a nestmate victim entrapped in an artificial snare. The data obtained for each of the four subgroups of ants are shown in ascending order. Red: Rescue behaviour directed towards the wire loop on the victim’s petiole. Yellow: Rescue behaviour directed towards the wire loop on the victim’s leg. Blue: Rescue attempts directed towards the victim’s body and to the substrate near the victim. WL− (*n* = 17): ants that engaged in rescue behaviour, but never directed their rescue attempts to any of the wire loops (WL) placed on the victim’s body; WL+ (*n* = 36): ants that engaged in various forms of rescue behaviour including also the responses to one or both wire loops placed on the victim’s body; WL+: L (*n* = 12): ants that responded only to the wire loop on the victim’s leg (L); WL+: L+P (*n* = 12): ants that responded to both wire loops, the one on the victim’s leg (L) and the one on the victim’s petiole (P); WL+: P (*n* = 12): ants that responded only to the wire loop on the victim’s petiole (P). *n* = number of individuals. Test duration: 20 min. The data presented here in the graphical form can also be found in the Tables S1, S3, S5 and S7 (Supplementary Online Materials).

**Figure 4:**
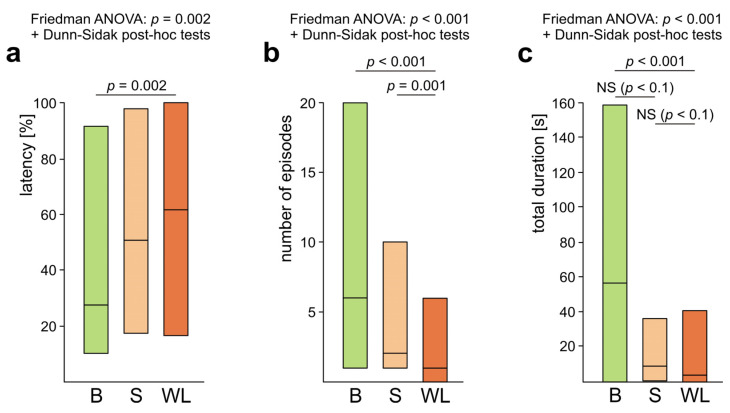
The values (medians and quartiles) of three variables quantifying three main subcategories of rescue behaviour displayed by workers of the red wood ant *Formica polyctena* in response to a nestmate victim entrapped in an artificial snare. Only the ants that engaged in rescue behaviour (*n* = 53) have been taken into account. (**a**) Latency from the start of the test to the first episode of the behaviour in question (expressed as the percent of the total test time). (**b**) Number of episodes of the behaviour in question recorded during the test. (**c**) Total duration of all episodes of the behaviour in question recorded during the test; B: Rescue attempts directed towards various parts of the victim’s body; S: Rescue attempts directed towards the substrate near the victim; WL: Rescue attempts directed towards the wire loops placed on the victim’s body. Test duration: 20 min. Statistics: Friedman ANOVA followed by Dunn–Sidak post hoc tests for pairwise comparisons of dependent data.

On an individual level, only about one third of the potential rescuers (53 out of 150 workers; 35.3%) engaged in rescue behaviour (Figure 3 and Figure 4, Tables S1–S8). The number of the ants that engaged in rescue behaviour during the same test ranged from one (on 9 tests) to the maximum possible number of five (on 2 tests). Participation of two workers in rescue activities took place on 8 tests and participation of three workers was observed on further 6 tests.

The tested ants showed important individual differences with respect to the degree of their general involvement in rescue attempts (Figure 3, Tables S1–S8). The total duration of all rescue attempts ranged from zero to 1097.58 s (91.5% of the total test time) (Figure 3, Table S3).

### 3.2. Occurrence of Various Subcategories of Rescue Behaviour

**Figure 5:**
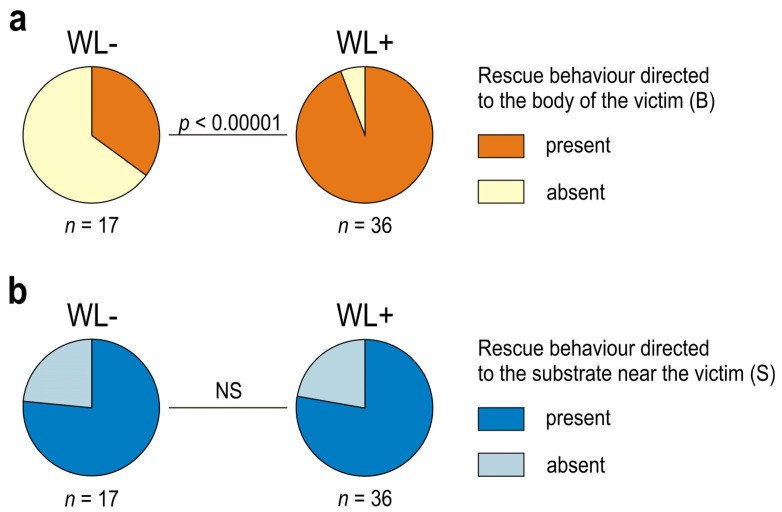
The rate of occurrence of two subcategories of rescue behaviour displayed by two subcategories of workers of the red wood ant *Formica polyctena* (WL− ants and WL+ ants) in response to a nestmate victim entrapped in an artificial snare. (**a**) Rescue behaviour directed towards various parts of the victim’s body (B). (**b**) Rescue behaviour directed towards the substrate (S) near the victim; WL−: ants that engaged in rescue behaviour, but never directed their rescue attempts to any of the wire loops (WL) placed on the victim’s body (*n* = 17); WL+: ants that engaged in various forms of rescue behaviour including also rescue attempts directed towards one or both wire loops placed on the victim’s body (*n* = 36). Test duration: 20 min. Statistics: two-tailed Fisher Exact Probability Test.

**Figure 6:**
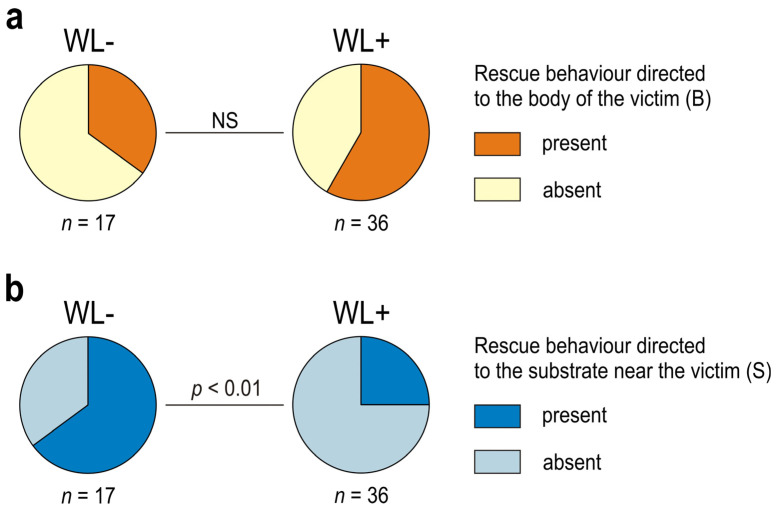
The rate of occurrence of two subcategories of rescue behaviour displayed by two subcategories of workers of the red wood ant *Formica polyctena* (WL− ants and WL+ ants) as the first rescue attempt directed towards a nestmate victim entrapped in an artificial snare. (**a**) Rescue behaviour directed towards various parts of the victim’s body (B). (**b**) Rescue behaviour directed towards the substrate (S) near the victim. Other explanations as in Figure 5.

**Figure 7:**
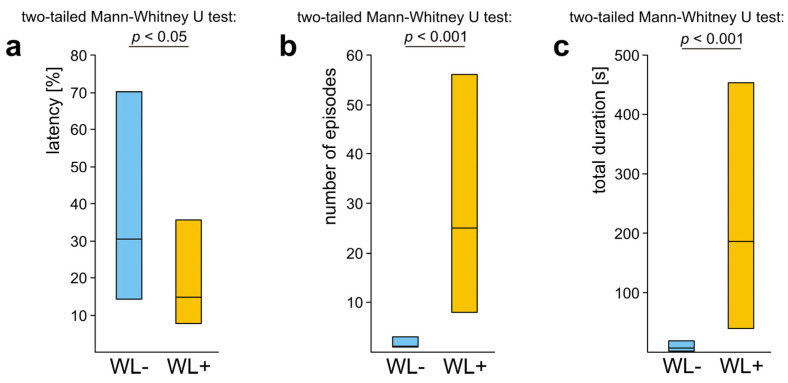
The values (medians and quartiles) of three main variables quantifying rescue behaviour (all subcategories pooled together) displayed by two subcategories of workers of the red wood ant *Formica polyctena* (WL− and WL+) in response to a nestmate victim entrapped in an artificial snare. (**a**) Latency from the start of the test to the first episode of rescue behaviour expressed as the percent of the total test time. (**b**) Number of episodes of rescue behaviour recorded during the test. (**c**) Total duration of all episodes of rescue behaviour recorded during the test. Statistics: two-tailed Mann−Whitney U test. Other explanations as in Figure 5.

**Figure 8:**
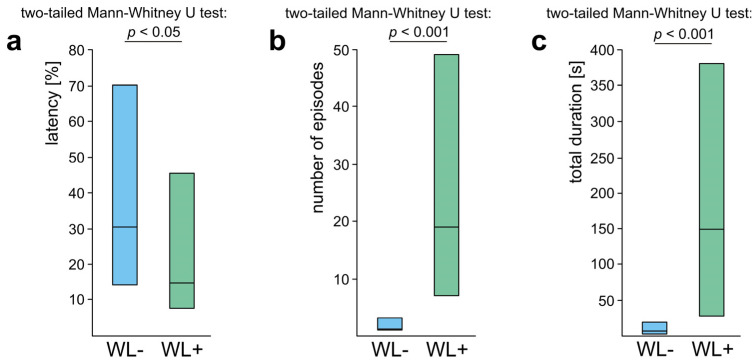
The values (medians and quartiles) of three main variables quantifying rescue behaviour (rescue attempts not involving responses to the wire loops placed on the victim’s body) displayed by two subcategories of workers of the red wood ant *Formica polyctena* (WL− and WL+) in response to a nestmate victim entrapped in an artificial snare. (**a**) Latency from the start of the test to the first episode of rescue behaviour expressed as the percent of the total test time. (**b**) Number of episodes of rescue behaviour recorded during the test. (**c**) Total duration of all episodes of rescue behaviour recorded during the test. Statistics: two-tailed Mann–Whitney U test. Other explanations as in Figure 5.

**Figure 9:**
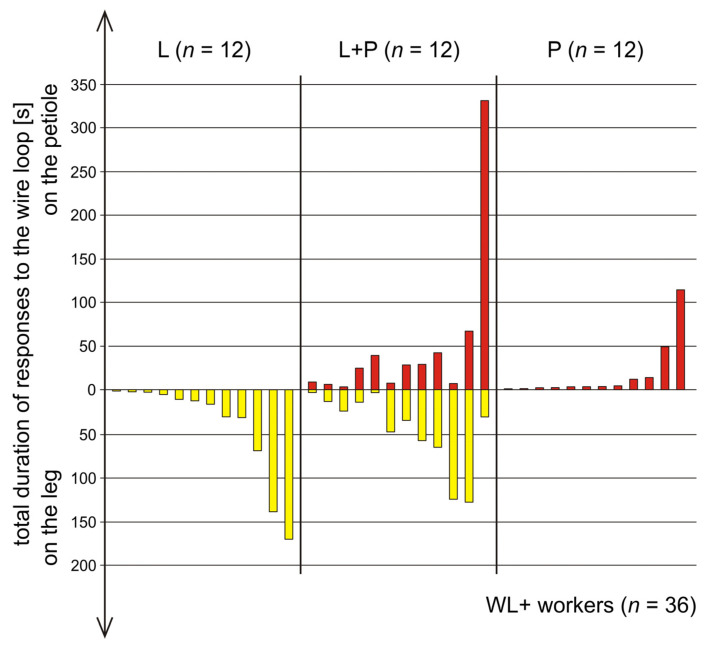
Total duration of responses directed by individual workers of the red wood ant *Formica polyctena* to the wire loops placed on the leg (yellow bars) and on the petiole (red bars) of a nestmate victim entrapped in an artificial snare. Only the ants that responded to wire loops (WL+ workers, *n* = 36) have been taken into account. L: ants that directed their rescue attempts only to the wire loop placed on the victim’s leg (L). L+P: ants that directed their rescue attempts to both wire loops, the one placed on the victim’s leg (L) and the one placed on its petiole (P). P: ants that directed their rescue attempts only to the wire loop placed on the victim’s petiole (P). The values of the total duration of responses directed towards the wire loop(s) are shown in ascending order in each ant subgroup. Test duration: 20 min. Statistics: Kruskal–Wallis ANOVA (*p* = 0.005) followed by Dunn–Sidak post hoc tests (P vs. LP: *p* = 0.003, L vs. LP and L vs. P: both NS). The data presented in this Figure in the graphical form can also be found in the Tables S3, S5 and S7 (Supplementary Online Materials).

**Figure 10:**
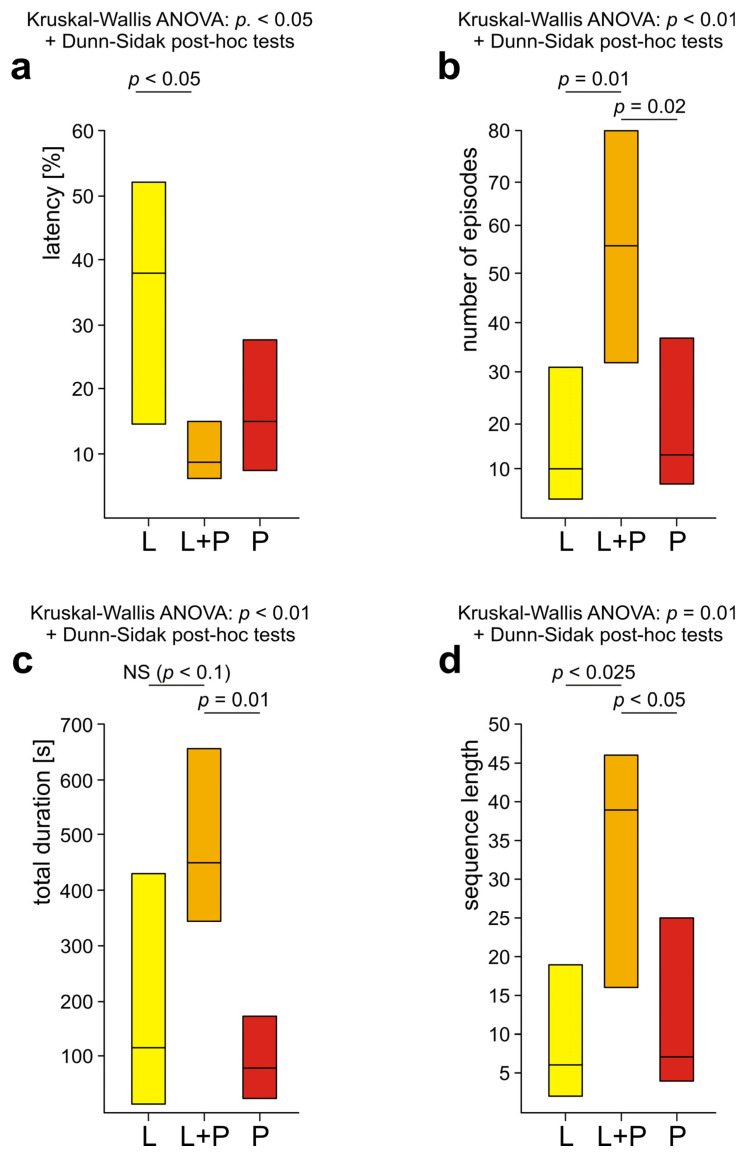
The values (medians and quartiles) of four variables quantifying rescue behaviour displayed by three subcategories of workers of the red wood ant *Formica polyctena* (L, L+P and P ants, *n* = 12 in the case of each ant group) in response to a nestmate victim entrapped in an artificial snare. (**a**) Latency from the start of the test to the first episode of rescue behaviour expressed as the percent of the total test time. (**b**) Number of episodes of rescue behaviour recorded during the test. (**c**) Total duration of all episodes of rescue behaviour recorded during the test. (**d**) Number of elements of the sequence of successive subcategories of rescue behaviour recorded during the test (responses to the victim’s body, to the substrate near the victim, to the wire loop on the victim’s leg and to the wire loop on the victim’s petiole). Other explanations as in Figure 9.

The tested ants showed important individual differences not only with respect to presence/absence of rescue behaviour, but also with respect to various features of that behaviour, and, in particular, with respect to their engagement in its different subcategories (Figure 3, Figure 4, Figure 5, Figure 6, Figure 7, Figure 8, Figure 9, Figure 10, Tables S1–S8). Rescue attempts directed towards the victim’s body (B), towards the substrate near the victim (S) and towards the wire loops placed on the victim’s body (WL) were recorded, respectively, in the case of 40, 41 and 36 ants, which corresponds to 75.5%, 77.4% and 67.9% of the ants that were observed to engage in rescue behaviour (*n* = 53).

The overall analysis carried out by means of Friedman ANOVA discovered significant differences in the case of all three variables calculated to quantify these three behavioural subcategories: the latency from the start of the test to the first episode of the behaviour in question, the number of episodes of that behaviour and its total duration (Figure 4a–c). Further analysis with the use of Dunn–Sidak post hoc tests revealed that rescue attempts directed towards the victim’s body (B) started to appear significantly more rapidly (Figure 4a), were significantly more numerous (Figure 4b) and had significantly longer total duration (Figure 4c) than those directed towards the wire loops (WL). Rescue attempts directed towards the wire loops (WL) were also significantly less numerous than those directed towards the substrate near the victim (S) (Figure 4b). Lastly, in the case of the total duration of various subcategories of rescue attempts the comparisons of B versus S and S versus WL discovered additionally two non-significant trends (Figure 4c). All these data taken together show that rescue attempts directed towards the wire loops (WL) represented the subcategory of rescue behaviour the least well expressed during the present experiment.

### 3.3. WL− Ants

About one third of the ants observed to engage in rescue behaviour (17 out of 53; 32.1%) did not direct their rescue attempts to any of the wire loops (WL) placed on the victim’s body (Figure 3, Tables S1 and S2). These WL− ants engaged solely in rescue attempts directed towards the victim’s body [(B); behaviour displayed by 6 workers (35.3% of the WL− ants)] (Figure 5a) and to the substrate near the victim [(S); behaviour displayed by 13 workers (76.5% of WL− ants)] (Figure 5b).

The majority of WL− ants (15 out of 17; 88.2%) employed only a single technique of rescue attempts and most frequently engaged solely in rescue behaviour directed towards the substrate near the victim (11 cases; 64.7% of WL− ants) (Table S2). Much less frequently, rescue attempts of WL− ants were directed exclusively to the victim’s body (4 cases; 23.5% of WL− ants) (Table S2). Lastly, only two WL− ants (11.8.% of the ants from that worker subcategory) directed their rescue attempts to both the victim’s body and the substrate near the victim (Table S2). Both these ants switched repeatedly between these two subcategories of rescue behaviour. However, sequences of successive subcategories of their rescue attempts were relatively short (consisted of only 7–8 elements) (Table S2).

Total durations of rescue attempts shown by WL− ants were relatively short, too, with a maximum value of 153.88 s (12.82% of the total test time) (Figure 3, Table S1).

The first episode of rescue behaviour observed during the tests with WL− ants consisted most frequently of rescue attempts directed towards the substrate near the victim (S) (11 out of 17 cases; 64.7% of WL− ants) (Figure 6b, Table S2).

### 3.4. WL+ Ants and Their Comparisons with WL− Ants

The majority of the ants observed to perform rescue behaviour (36 out of 53; 67.9%) engaged in biting/pulling of one or both of the wire loops (WL) placed on the body of the victim and, therefore, have been labelled as WL+ ants (Figure 3 and , Figure 5, Figure 6, Figure 7, Figure 8, Figure 9, Figure 10, Tables S3–S8). As already pointed out, exactly the same number of WL+ ants (*n* = 12) engaged in biting/pulling of the wire loop on the victim’s leg (L ants), on the victim’s petiole (P ants) and of both loops (L+P ants).

Whereas WL− ants usually engaged only in a single subcategory of rescue behaviour (Table S2), in the case of WL+ ants such a situation took place only once: one worker from the subgroup L performed only a single response to the wire loop on the victim’s leg (Figure 3, Table S4). All the remaining WL+ ants (35 individuals; 97.2%) apart from directing their rescue behaviour to the wire loop(s) placed on the victim’s body (Figure 3 and Figure 9, Tables S3–S8) also engaged in other forms of rescue behaviour (Figure 3, Figure 5, Figure 6, Figure 8, Tables S3–S8). These rescue attempts were directed most frequently to the victim’s body (B) (34 workers; 94.4% of WL+ ants), but only slightly less frequently to the substrate near the victim (S) (28 workers; 77.8% of WL+ ants).

WL+ ants differed significantly from WL− ones with respect to the rate of occurrence of rescue behaviour directed towards the victim’s body (B) (Figure 5a). That subcategory of rescue behaviour was observed in the majority (94.4%) of the WL+ ants (94.4%), but only in about one third (35.3%) of the WL− ants. However, the rate of occurrence of rescue attempts directed towards the substrate near the victim (S) was high in both WL− and WL+ ants (76.5% and 77.8%, respectively) and did not differ between these two ant subcategories (Figure 5b).

The first episode of rescue behaviour performed by the WL+ ants consisted most frequently of rescue attempts directed towards the victim’s body (B) (21 out of 36 cases; 58.3%) (Figure 6a), differently than in the case of WL− ants that most frequently started their rescue attempts from responses directed towards the substrate near the victim (S) (Figure 6b). However, when only the first episodes of rescue behaviour were taken into account, WL− and WL+ ants did not differ significantly with respect to the rate of occurrence of rescue attempts directed towards the victim’s body (Figure 6a), differently than in the analysis in which all instances of that behaviour were taken into account (Figure 5a). An opposite situation occurred in the case of rescue attempts directed towards the substrate near the victim (S): WL− ants differed significantly from WL+ ants when only the first episodes of their rescue behaviour were taken into account (Figure 6b), but no significant differences between WL− and WL+ ants were observed when all instances of that behaviour were taken into account (Figure 5b).

Lastly, only in the case of six WL+ ants (16.7%) the first episode of rescue behaviour consisted of rescue attempts directed towards one of the wire loops. Such a situation was recorded in the case of three L ants, two L+P ants (both responses were directed towards the wire loop on the victim’s petiole) and one P ant (Tables S4, S6 and S8).

WL+ ants also differed significantly from the WL− ones with respect to the values of three main variables calculated to quantify their overall rescue behaviour (all subcategories pooled together). WL+ ants started to engage in rescue behaviour after a shorter latency from the start of the test (Figure 7a), engaged in a higher number of episodes of that behaviour during the test (Figure 7b) and devoted more time to rescue attempts (Figure 7c).

As significant differences between WL− ants and WL+ ones revealed by the analysis of the variables quantifying their overall rescue behaviour (Figure 7) might have been related simply to the fact that WL+ ants had a richer repertory of subcategories of rescue behaviour than WL− ones, we subsequently compared rescue behaviour of these two subgroups of ants taking into account only rescue attempts not involving the responses to the wire loops (Figure 8). However, this analysis yielded identical results as the previous one. This time, too, WL+ ants started to engage in rescue behaviour after a shorter latency from the start of the test (Figure 8a), performed a higher number of episodes of that behaviour during the test (Figure 8b) and devoted more time to that behaviour (Figure 8c) than WL− ones.

These findings imply that the differences between WL− ants and WL+ ones with respect to their general propensity to engage in rescue behaviour cannot be attributed to the fact that WL+ ants had a richer repertory of rescue behaviour patterns including also the responses to the wire loops. Evidently, WL+ ants differed from WL− ones not only with respect to presence/absence of rescue attempts directed towards the wire loops. WL+ ants also showed much higher readiness than WL− ones to engage in rescue behaviour not involving the responses to the wire loops.

Sequences of various subcategories of rescue behaviour [rescue attempts directed towards the victim’s body (B) and the substrate near the victim (S), and in the case of WL+ ants also towards the wire loop on the victim’s petiole (P) and the wire loop on its leg (L)] were also significantly longer (two-tailed Mann–Whitney U test: *p* < 0.0001) in the case of WL+ ants than in the case of WL− ones. Whereas medians, quartiles and range of that variable obtained for WL− ants were equal to 1, 1–1 and 1–8, respectively, in the case of WL+ ants these values were equal to 15, 5–39 and 1–75, respectively (see also Tables S2, S4, S6 and S8).

### 3.5. Comparison of Rescue Behaviour Performed by Workers from Various Subgroups of WL+ Ants (L, L+P and P)

WL+ ants did not only differ in many respects from WL− ants (Figure 5, Figure 6, Figure 7 and Figure 8), but they were also far from being homogenous with respect to worker behaviour. Ants from three subgroups of WL+ ants (L, L+P and P) differed not only with respect to the type of the wire loops to which they have responded, but also with respect to many other features characterizing their rescue behaviour (Figure 3, Figure 9 and Figure 10, Tables S3–S8).

The behaviour of the ants belonging to these three subgroups showed important individual differences with respect to the total duration of responses to the wire loops (Figure 3 and Figure 9, Tables S3, S5, S7). In particular, P ants that responded only to the wire loop on the victim’s petiole (P) acting as a snare and, therefore, might be expected to represent the subgroup of rescuers characterized by the most advanced and most precisely targeted rescue behaviour, proved to be less active as rescuers than the ants from the remaining two subgroups of WL+ ants (Figure 3, Figure 9 and Figure 10, Tables S3–S8). The ants from the subgroup L+P were the most active both in responding to the wire loops (Figure 3 and Figure 9, Table S5) and with respect to their general engagement in rescue activities (Figure 3 and Figure 10, Tables S3–S8). The total duration of responses to the wire loops recorded in L+P ants was significantly higher than in the case of P ants (Figure 9). L ants behaved in a way intermediate with respect to both P and L+P ants and did not differ significantly from workers from any of these two subgroups (Figure 9).

The analyses of four variables quantifying overall rescue behaviour of three subgroups of WL+ ants (the latency from the start of the test to the first episode of rescue behaviour, the number of episodes of rescue behaviour recorded during the test, the total duration of that behaviour and the number of elements of the sequence of successive subcategories of rescue behaviour) discovered significant inter-group differences in the case of all these variables (Figure 10a–d). These results fully confirmed that L+P ants were the most active as rescuers. As revealed by the post hoc tests, L+P ants engaged in a significantly higher number of bouts of rescue behaviour and performed significantly longer sequences of successive behavioural subcategories than both L and P ants (Figure 10b,d). The total duration of rescue behaviour of L+P ants was also significantly longer than in the case of P ants, and although the comparison of L+P ants with L ones revealed only a non-significant trend, it was very close to significant (*p* = 0.057) (Figure 10c). The latency from the start of the test to the first episode of rescue behaviour was also the shortest in the case of L+P ants. However, the differences between the values of that variable obtained for various subgroups of WL+ ants proved to be significant only in the case of the comparison of L+P ants with L ones. P ants started to engage in rescue behaviour equally rapidly as L+P ants (Figure 10a). The ants that directed their rescue attempts to only one type of a wire loop placed on the victim’s body (L ants and P ants) in the majority of the cases started to engage in rescue behaviour by directing their rescue attempts to the body of the victim [L ants: 7 cases; 58.3%; P ants: 9 cases (75.0%)]. Only two ants in each of these subgroups (16.7%) started to engage in rescue behaviour by directing their rescue attempts to the substrate near the victim. Finally, three L ants (25.0%) and one P ant (8.3%) started their rescue activities by responding to the wire loop on the victim’s leg and on the victim’s petiole, respectively (Tables S4 and S8). 

In contrast, the ants that responded to both wire loops (L+P ants) equally frequently (in 5 cases; 41.7%) directed their first episode of rescue behaviour to the victim’s body and to the substrate near the victim. In the remaining two cases (16.7%), the first episode of their rescue behaviour was directed towards the wire loop on the victim’s petiole. No ant from that group started to rescue the victim by responding to the wire loop on its leg (Table S6).

Lastly, the responses to the body of the victim were performed by all L+P ants, all P ants (100.0% in both cases) (Tables S6 and S8) and the majority of L ants (10 cases; 83.3%) (Table S4). The responses to the substrate near the victim were slightly less frequent, but also very common. They were performed by 75.0% of both L and P ants (nine workers from each of these groups; Tables S4 and S8) and 83.3% of L+P ants (ten workers; Table S6). These findings fully confirm that the individuals responding to the wire loops engage also very readily in other, simpler forms of rescue behaviour.

### 3.6. Comparison of Rescue Behaviour Directed towards the Wire Loop on the Victim’s Leg (L) and on Its Petiole (P)

The comparisons of the values of three variables quantifying biting/pulling behaviour directed by WL+ ants towards the wire loops placed on the victim’s leg (L) and on its petiole (P) (the latency from the start of the test to the first episode of the analysed behaviour, the total number of episodes of that behaviour recorded during the test and the total duration of all episodes of that behaviour) (Figure 3, Figure 9, Figure 10, Tables S3, S5 and S7) did not discover any significant differences (Table 2A). In other words, the ants did not show preference for the wire loop on the victim’s petiole (P) acting as a snare implicated in the victim’s entrapment.

As already pointed out, *Formica polyctena* workers are known to respond to small moving objects with attraction and biting [107,117]. Therefore, it could not be *a priori* excluded that movements of the leg bearing the wire loop played an important role in triggering the responses of the tested ants to that loop. This in turn might have masked the preference of the rescuer ants for the loop on the petiole resulting from recognition of its significance for the victim’s entrapment. In other words, our failure to detect the preference for the wire loop on the victim’s petiole might have resulted from the fact that such a preference did indeed arise as consequence of sophisticated cognitive abilities, but was counterbalanced and masked by simultaneous propensity of the tested ants to approach and bite small moving objects. 

To shed more light on that question, we carried out an additional analysis in which we compared the responses of WL+ ants to two wire loops placed on the victim’s body after having discarded from analysis all responses to the loop on the victim’s leg that were initiated while that leg was in movement. However, this time, too, the values of the variables calculated to quantify the responses of the ants to the wire loops placed on the victim’s leg (L) and petiole (P) did not show significant differences (Table 2B). 

Absence of differences between the results of analyses in which we took into account all rescue attempts directed towards the wire loops on the victim’s leg (Table 2A) and only those of them that were carried out while the leg bearing the loop was immobile (Table 2B) is not surprising, as responses to the wire loop on the victim’s leg initiated when that leg was in movement (LM) proved to be very infrequent. Only six ants engaged in that behaviour during the whole experiment and only one ant engaged in it more than one time (Table 3). Moreover, the response to the wire loop on the victim’s leg initiated while that leg was moving was always preceded by other subcategory of rescue behaviour (Table 3). This last finding clearly implies that rescue behaviour of these ants was not initiated as a response to movements of the loop on the victim’s leg.

We also checked if responses of the tested *Formica polyctena* workers to the wire loops on the victim’s leg (L) and on its petiole (P) were influenced by different length of the parts of these wire loops accessible for the rescuers. Longer lengths of the pieces of wire used to form the loops on the legs of the victims might have enhanced the frequency and duration of episodes of rescue behaviour directed towards these loops and that in turn might have masked the preference for the wire loop on the victim’s petiole arising from recognition of its crucial role in the victim’s entrapment. However, measurements of parts of wire loops exposed to the rescuers did not discover significant differences between the loops placed on the legs and on the petioles of the victims (Table 4). This finding allows us to conclude that responses of the tested ants to wire loops placed on the victim’s body were not influenced by differences in length of parts of these loops accessible for the rescuers.

Absence of preference for the wire loop on the victim’s petiole is also illustrated by several other findings. First, as already mentioned, only very infrequently (in 3 cases during the whole experiment) the first episode of rescue behaviour performed by the ant consisted of rescue attempts directed towards the wire loop on the victim’s petiole (Tables S6 and S8). Second, the tested ants equally frequently started their rescue behaviour from responses to the wire loop placed on the victim’s petiole (3 cases; Tables S6 and S8) and on the victim’s leg (3 cases; Table S4). Third, in the case of the ants from the L+P subgroup the first response to any of the wire loops was directed with similar frequency to the loop on the victim’s leg and to the loop on the victim’s petiole (5 and 7 cases, respectively; Table S6). Fourth, only one L+P ant responded first to the loop on the victim’s leg and then switched to rescue attempts directed towards the loop on the victim’s petiole and returned no more to responding to the loop on the victim’s leg (Table S6). Such behaviour might suggest that that particular ant identified the wire loop on the victim’s petiole as the correct target of its rescue activities. However, such a sequence of responses to the wire loops was observed only a single time during the whole experiment. Moreover, a single case of an opposite situation (a single switching from rescue attempts directed towards the wire loop on the victim’s petiole to those directed towards the wire loop on the victim’s leg) was also observed (Table S6). All remaining L+P ants (10 out of 12 cases; 91.3%) kept to switch repeatedly (up to 21 times) between the responses to both loops (Table S6). Lastly, no ant responded solely to the wire loop on the victim’s petiole without engaging in other forms of rescue behaviour (Figure 3, Tables S3–S8).

## 4. Discussion

### 4.1. The Most Important Novel Aspects of Our Methods and Findings

Our present experiment allowed us to shed more light on causal factors underlying ant rescue behaviour and, in particular, precisely targeted rescue behaviour. This was possible thanks to the application of a novel version of the nestmate rescue test consisting of a confrontation of potential rescuers with a nestmate victim entrapped in an artificial snare. In contrast to earlier studies with the use of the artificial snare bioassay [29,59,61,62,64,65,66,67,68,69,70,72,73,74,75,76,77], in our study each nestmate victim was bearing on its body not just one, but two wire loops, one placed on its petiole and an additional one placed on its leg. Only the loop on the victim’s petiole was acting as a snare, the loop on the leg was not implicated in the victim’s entrapment. We asked if the tested ants are indeed able to identify correctly the object responsible for the victim’s entrapment and then direct their precisely targeted rescue behaviour to that particular object and not to another similar wire loop playing no role in restraining the victim.

Our findings clearly demonstrated that the tested *Formica polyctena* workers did not preferentially direct their rescue attempts to the wire loop acting as a snare, but responded in the same way to both wire loops placed on the victim’s body. Moreover, we found out that rescue behaviour of the tested ants did not consist predominantly of precisely targeted rescue attempts: almost all ants that have responded to the wire loops engaged also in rescue attempts directed towards the victim’s body and to the substrate near the victim. Actually, rescue attempts directed towards the wire loops represented the least common subcategory of rescue attempts and the overall strategy adopted by the most active rescuers consisted of frequent switching between various subcategories of rescue behaviour. 

We also checked if rescue behaviour of the tested workers was influenced by movements of the leg bearing the additional wire loop and by the differences in length of the parts of the wire loops accessible to the rescuers. Neither of these factors was found to contribute in a significant way to our present findings.

### 4.2. Occurrence of Rescue Behaviour and Its Subcategories

The results of our present study fully confirmed earlier findings showing that some workers of the red wood ant *Formica polyctena* engage in rescue behaviour in response to a nestmate victim entrapped in an artificial snare or captured by an antlion larva [66]. 

Various subcategories of rescue behaviour recorded in the present study, such as biting/pulling of various parts of the victim’s body, responses to the substrate near the victim (sand digging, removal of small pebbles, responses to the paper disc acting as a part of the snare apparatus) and biting and pulling of the wire loops placed on the victim’s body were also already described in numerous studies investigating ant rescue behaviour [28,29,59,60,61,62,63,65,66,67,68,72,74,75,76,77].

The most frequently observed subcategory of rescue behaviour performed by *Formica polyctena* workers during our experiment consisted of rescue attempts directed towards various parts of the victim’s body. Similar finding was obtained for workers of *Formica cinerea*, a sand-dwelling ant species from the same genus, *Formica* [60,74]. However, in the case of another sand-dwelling species, *Cataglyphis piliscapa*, the most common subcategory of rescue behaviour consisted of digging around the victim [68]. Interestingly, workers of another species from that genus, *Cataglyphis nigra*, engaged preferentially in digging around the victim when coming at the rescue of adult ants entrapped in snares, but preferred to engage in pulling behaviour when responding to nestmate pupae held by similar snares [77]. As pointed out by the authors of that study, the tested ants adapted their rescue behaviour to specific requirements of different victims. Whereas trapped adults may rescue themselves when receiving moderate help and do not require pulling, pupae may be more easily squeezed out of tight spots owing to their softer cuticle.

Somewhat surprisingly, responses to the wire loops placed on the victim’s body represented the least well expressed subcategory of rescue behaviour displayed by *Formica polyctena* workers tested in this study. This finding highlights relatively limited importance of precise identification of the source of the victim’s restraint in the mediation of rescue behaviour observed during our present experiment.

### 4.3. Behavioural Profiles of Formica Polyctena Workers

*Formica polyctena* workers tested in the present experiment showed important individual differences with respect to many aspects of their behaviour. First, although rescue activities were observed on the majority of the tests (83.3%), only about one third of the potential rescuers actually engaged in rescue attempts. Similar phenomenon (participation of a relatively small part of the potential rescuers in rescue attempts) was repeatedly reported in earlier studies of ant rescue behaviour [29,59,60,61,62,63,65,70,74,75,76,77]. In some studies only a few individuals were observed to engage in rescue actions. For instance, in a field study investigating rescue behaviour of harvester ants from the species *Veromessor pergandei* on average only six out of thousands of workers passing close to the spider web containing a trapped nestmate engaged in rescue behaviour [78].

In the aforementioned study investigating rescue behaviour of *Formica polyctena* workers with the use of two bioassays, antlion larva capture bioassay and artificial snare bioassay [61], rescue behaviour was performed by less than 20% of workers tested in each bioassay and, thus, its rate of occurrence was even lower than in the case of our present experiment. It is thus not surprising that in a subsequent review [20] rescue behaviour of *Formica polyctena* workers was classified as „detected but weak and/or infrequent” (however, with an additional remark that the propensity of these ants to engage in rescue activities may be underestimated) [20].

However, we should bear in mind that such a relatively low rate of occurrence of rescue behaviour was recorded in *Formica polyctena* workers tested with the use of dyadic nestmate rescue tests consisting of a confrontation of a victim with only one potential rescuer [61]. In contrast, the bioassay used in our study consisted of a confrontation of a victim with five potential rescuers. Such a number of potential rescuers was chosen on the basis of recommendations provided in the first study investigating ant rescue behaviour with the use of artificial snare bioassay [29]. As reported by the authors of that study, preliminary tests with the use of that bioassay strongly suggested that at least five potential rescuers must be present to evoke rescue behaviour in workers of *Cataglyphis piliscapa*. As a consequence, artificial snare bioassay consisting of a confrontation of five potential nestmate rescuers with a single victim was also used in several further studies investigating ant rescue behaviour [64,65,66,68,72,74] and the number of potential rescuers used in bioassays investigating ant rescue behaviour was sometimes even higher (up to 10 individuals) [73]. At the same time other studies demonstrated, however, that rescue behaviour may also be expressed during dyadic nestmate rescue tests [43,59,60,61,62,66,68,74,75,76]. Nevertheless, as demonstrated by a more recent study [74], the number of potential rescuers exerts an important impact on the propensity of ant workers to engage in rescue behaviour. Worker group size and the number of individuals present together in an experimental arena have also been shown to exert an important impact on other behaviour patterns shown by *Formica polyctena* workers and, in particular, on their responses to potential insect prey and to brood [109,110,111,112,115]. Therefore, final estimation of propensity of *Formica polyctena* workers to engage in rescue behaviour requires further experimental work that should investigate also context-dependence of that propensity.

We should also bear in mind that rescue activities do not belong to the behavioural repertoires of all colony members. As pointed out by several researchers of ant rescue behaviour, whereas some individuals engage in rescue activities, other individuals, the non-rescuers, actively refrain from rescue attempts. Upon a confrontation with a trapped nestmate, such non-rescuers immediately withdraw from it and leave rescuers unrestricted access to the victim [66,72]. These observations found full confirmation in the results of a study involving the use of genetic methods [72]. As revealed by that study, in societies of *Cataglyphis piliscapa* propensity to engage in rescue behaviour is a heritable behavioural specialization and behaviour of non-rescuers also has genetic correlates.

Second, during the present experiment, the ants that engaged in rescue activities did not form a subgroup homogenous with respect to behaviour. About one third of these rescuers, the WL− ants, never directed their rescue attempts to any of the wire loops placed on the victim’s body. WL− ants were also very little active as rescuers: their rescue behaviour usually consisted of a single episode and the total duration of their rescue activities only in one case exceeded one tenth of the total test time. It also may be noted that rescue behaviour of WL− ants consisted predominantly of sand digging and other responses to the substrate near the victim and not of rescue attempts directed towards the victim’s body.

In contrast, the ants that were observed at least once to direct their rescue attempts towards one of the wire loops placed on the victim’s body (WL+ ants) were much more active as rescuers. They started their rescue activities earlier than WL− ants, performed more episodes of that behaviour, devoted more time to it and switched more frequently between various subcategories of rescue behaviour.

WL+ ants also showed a higher propensity to direct their rescue behaviour to the victim’s body than WL− ants. This suggests that WL+ ants might have responded to the wire loops placed on the victim’s body at least partly as a continuation of their responses to various parts of the victim’s body. Such a possibility is also supported by the fact that rescue attempts directed towards the wire loop on the victim’s petiole were almost always, in the case of 22 out of 24 ants that engaged in that behaviour (91.7%), preceded by rescue activities directed towards the victim’s body.

It also should be stressed that WL+ ants differed from WL− ones not only with respect to presence/absence of rescue attempts directed towards the wire loops on the victim’s body, but also showed higher readiness to engage in rescue behaviour not directed towards the wire loops. In other words, engagement in rescue behaviour directed towards the wire loops was found to be accompanied by increased (and not decreased) readiness to engage in other forms of rescue behaviour.

Third, our study also revealed the existence of important behavioural differences between three subgroups of WL+ ants; namely, the ants that responded solely to the wire loop on the victim’s leg (L ants), the ants that have responded to both wire loops on the victim’s body (L+P ants) and the ants that have responded solely to the wire loop on the victim’s petiole (P ants). Somewhat surprisingly, L+P ants were the most active as rescuers and P ants were the least active. That last finding will be discussed in more detail in the Section 4.5.

Fourth, important individual differences were also detected within each of these ant subgroups. In particular, relatively numerous individuals were relatively little active as rescuers. Nevertheless, in the analyses carried out in the present study we took into account all individuals observed to engage in rescue behaviour, similarly as in the majority of studies investigating ant rescue behaviour. However, we may note that in some studies a different approach has been applied. Some ant species in which rescue behaviour was observed infrequently have been classified as species not engaging in rescue behaviour [59,61,67] and in some studies only the ants that met a strictly defined criterion (for instance, performed one or more of the three rescue behaviour patterns for at least 60 s within the 4-min test) [72] were classified as confirmed rescuers [66,68,72]. Such criteria allowed the researchers to focus attention on the individuals engaging in rescue behaviour as a consequence of a specific behavioural specialization [11,19,20,30,64,68,72,77]. Unfortunately, the criteria used to tell apart the confirmed ant rescuers from the non-rescuers were to some degree arbitrary and differed not only between different studies [66,68,72], but even between different experiments carried out within the same study [66]. Moreover, it should be remembered that the approach applied in our present study (focusing attention on all individuals that were observed to engage in rescue behaviour without discarding less active ones) also has merits, as it allows the researchers to take into account the whole ranges of variability of the analysed behavioural traits.

### 4.4. Absence of Preference for the Wire Loop Acting as a Snare

The results of our experiment revealed that *Formica polyctena* workers did not direct their rescue behaviour preferentially towards the wire loop on the victim’s petiole that acted as a snare responsible for the victim’s restraint. We were also able to reject the hypothesis that absence of preference for the wire loop on the victim’s petiole resulted from joint involvement of two antagonistic causal factors: the propensity of the rescuers to precisely target their rescue behaviour to the wire loop identified by them as the object crucially responsible for the victim’s restraint and their propensity to approach and bite small moving objects [107,117]. Similarly, we were also able to reject the hypothesis that the responses of the tested ants to two categories of wire loops placed on the victim’s body were influenced by differences in the length of the fragments of wire used to form the parts of the loops easily accessible for the rescuers.

Absence of preference for the wire loop acting as a snare over the one placed on the victim’s leg strongly suggests that responses of the tested ants to the wire loops must not necessarily have involved highly advanced cognitive processes that allowed the rescuers to identify the object responsible for the victim’s restraint and to respond to it by precisely targeted rescue behaviour. Moreover, we also found out that, with a single exception, all ants that had responded to the wire loops also engaged in other forms of rescue behaviour and that rescue attempts directed towards wire loops represented the least common subcategory of rescue activities. In other words, precisely targeted rescue behaviour, even if present, was usually not predominant.

These findings cast doubt on the importance of the proposed causal relationship between the responses of the ant rescuers to the wire loop acting as a snare and hypothetical highly advanced cognitive processes allowing them to achieve precise identification of the object responsible for restraining the movements of the victim. In light of our present findings it seems rather probable that responses of *Formica polyctena* workers to the wire loops placed on the victim’s bodies represented largely their efforts to remove foreign objects from the surface of the victim’s body. Removal of foreign bodies from the body surface of other individuals is very well documented in ants. Ants from many species and subfamilies were reported to engage in allogrooming (grooming other individuals) to remove from the bodies of nestmate adults and brood a wide range of undesirable objects including conidia of entomopathogenic fungi [127,128,129,130,131,132,133,134,135,136,137,138,139,140,141,142,143,144,145,146,147,148], animal parasites such as nematodes and cestodes [127,130,149,150], spiderwebs [71,78] and inanimate materials such as tracer dyes [151], talcum powder [147] and paint colour markings [48,152]. Such behaviour was also reported in the ant species investigated in the present study. Workers of *Formica polyctena* were observed to respond by allogrooming to their nestmates that had been treated with bacterial endotoxin to activate their immune systems in a way similar to that occurring during infections [153].

One of the recent review papers devoted to ant rescue behaviour [20] presented an opinion that biting of the snare does not represent a response to a foreign object because empty nylon snares were met with total indifference by *Cataglyphis piliscapa* workers investigated in the first study with the use of artificial snare bioassay [29]. However, ant responses to specific stimuli and/or treatments are usually strongly context-dependent [108,109,110,111,121,154,155,156]. Therefore, absence of responses to an empty snare does not automatically stand in contradiction with the possibility that the same ants will engage in intense attempts to remove such an object when it will adhere to the body surface of a nestmate.

We would also like to stress that we cannot exclude the possibility that our present findings might have resulted from joint action of several causal factors intervening in the mediation of rescue behaviour of the tested *Formica polyctena* workers. As already told, thanks to our additional analyses we were able to exclude the possibility that their rescue behaviour was significantly influenced by their attraction to small moving objects and by the effects of differences in the length of the fragments of wire loops accessible for the rescuers. However, we cannot exclude that causal factors driving their rescue behaviour involved both the urge to remove foreign objects from the body surface of the victim and the ability to identify the object responsible for the victim’s restraint, as these two factors are not mutually exclusive. We may also observe that the wire loop on the victim’s leg is placed more distally than the wire loop passing over the victim’s petiole and, therefore, may be more easy to be perceived by the approaching potential rescuer. This factor might also have influenced the behaviour of the tested ants.

We should also remember that absence of preferences manifesting themselves as different responses to different stimuli does not automatically imply that the ants do not discriminate between such stimuli. For instance, workers of the carpenter ant *Camponotus floridanus* were evidently able to tell familiar non-kin apart from familiar sisters, as the latter were antennated and attacked less frequently than familiar non-kin workers. However, workers tested in that study did not show preferences for either of these two subcategories of nestmates in the context of food exchange and grooming [156]. Therefore, the results of our present study allow us only to draw a conclusion that *Formica polyctena* workers tested in our experiment did not prefer to direct their rescue behaviour to the wire loop acting as a snare, but they do not allow us to state with certainty that the tested ants did not discriminate between the wire loops involved and not involved in the victim’s entrapment.

We also may ask if the ants tested by means of our novel bioassay could indeed be able to discriminate between the wire loop acting as a snare and the other one not involved in the immobilization of the victim. The ants tested in this study only exceptionally started to direct their rescue behaviour to the loop on the victim’s leg while that leg was in movement and when the loop on the leg was immobile, it was similar with respect to its mobility to the wire loop acting as a snare. However, biting and/or pulling of the loop on the victim’s leg was usually followed by movements of that loop and the leg that bore it. In other words, even if the responses to the loop of the leg have been initiated when the leg was immobile, the loop usually started to move as a consequence of biting/pulling behaviour of the rescuer. This in turn could allow the rescuer to detect the differences between the snare responsible for immobilization of the victim and the additional loop not involved in its entrapment. Nevertheless, our findings clearly demonstrated that even if the tested *Formica polyctena* workers were able to identify precisely the object responsible for the entrapment of the victim, this ability did not have its counterpart on behavioural level. Whatever were the exact causal factors underlying such behaviour, the ants did not direct their rescue activities preferentially to the wire loop responsible for the entrapment of the victim, but responded in a similar way to both loops present of the victim’s body. Moreover, the ants that engaged in precisely targeted rescue behaviour did not limit their rescue activities to responses to the wire loop acting as the snare, but engaged also in other subcategories of rescue behaviour. Actually, responses to the wire loops represented the least frequently performed subcategory of rescue activities.

### 4.5. Behavioural Profiles of the Most Active and the Least Active Rescuers

Rescue behaviour directed towards the wire loop on the victim’s petiole represents the most precise response of the rescuers to the problem encountered by the victim, as only that loop was involved in the victim’s immobilization. However, the ants that directed their rescue behaviour to that loop, but never directed it to the loop on the victim’s leg (P ants), were not the most active as rescuers. On the contrary, ants from this subgroup were the least active in engaging in rescue activities in comparison with all other ants that responded to the wire loops (WL+ ants).

The most active rescuers were found mostly among the ants that directed their rescue attempts to both wire loops present on the victim’s body (L+P ants). L+P ants were observed to start to engage in rescue behaviour most rapidly, to perform the highest number of episodes of that behaviour, to devote most time to rescue attempts and to switch most frequently between various subcategories of rescue behaviour. Moreover, the most active rescuers did not limit their rescue efforts to responses to the wire loops, but engaged in diverse other subcategories of rescue behaviour including pulling at various parts of the victim’s body, removing sand from the vicinity of the victim and responding to the paper disc to which the victim was tied. In other words, the overall strategy adopted by the most active rescuers of *Formica polyctena* did not consist of rescue attempts targeted precisely to the source of the victim’s problem, the snare on its petiole, but consisted of intense versatile switching between various subcategories of rescue behaviour.

Interestingly, L and P ants did not differ from L+P ants in a fully symmetrical way. Thus, three variables quantifying rescue behaviour (the number of episodes, the total duration of all episodes and the number of elements of the sequence of successive subcategories of rescue behaviour) took the highest values in the case of L+P ants, whereas L ants and P ants did not differ from each other in these respects. However, the highest values of the latency from the start of the test to the first episode of rescue behaviour were obtained for L ants, whereas L+P ants and P ants did not differ from each other. Moreover, only P ants differed significantly from L+P ants with respect to the total duration of rescue attempts (significantly lower in the case of P ants). These findings show thus that different variables quantifying the same behavioural subcategory were not simply correlated, as their analysis yielded results that did not simply mirror each other and that quantification of ant rescue behaviour with the use of a lesser number of variables might have led to loss of some interesting results.

It should, however, be remembered that the loops placed on the victim’s body were made of wire, and, therefore, ant rescuers were unable to bite through the snare or to tear it. Therefore, it cannot be excluded that the tendency of the most active rescuers to switch repeatedly between various alternative subcategories of rescue attempts might have been at least partly related to the fact that their attempts to break the snare were unsuccessful. Switching to another subcategory of rescue behaviour after unsuccessful attempts to break the snare was already reported in an earlier paper [19] in which the authors described how after futile attempts to bite through the snare some ant rescuers crawled underneath the filter paper to which the victim was tied and started to bite at the knot of the snare [see also the next Section 4.6].

### 4.6. Other Results Shedding Light on Cognitive Aspects of Rescue Behaviour of Workers of Formica Polyctena

Some other details of rescue activities observed during the present experiment also have important implications for better understanding of cognitive processes involved in ant rescue behaviour. In particular, during the whole experiment, we did not observe any instance of pulling of the victim’s antenna. Absence of antenna pulling was already reported in several earlier studies investigating rescue behaviour of ants from another formicine species, *Cataglyphis piliscapa* and interpreted in terms of avoidance of serious injuries of the victim (antennae were described as “fragile” and as “highly sensitive appendages that could be injured easily”) [29,68,72].

We do not possess any evidence allowing us to attribute the absence of antenna pulling during rescue attempts to cognitive processes involving some form of insight into the possible unfavourable consequences of that behaviour for the victim. However, absence of antenna pulling observed together with the concomitant presence of leg pulling must have had some underlying causal factors. The rescuers were evidently able to perceive the difference between the victim’s antennae and its legs, probably on the basis of some chemical cues, or as a consequence of perception of some other signals emitted by the victim. Thus, the ability to discriminate between the nestmate antennae and legs and to direct rescue activities only to the latter is also an important element of cognitive abilities of *Formica polyctena* workers and causal factors contributing to that ability are worth further studying in the future.

However, it should be remembered that absence of antenna pulling during rescue activities was not universally reported in all tested ant species. Czechowski et al. (2002) [28] recorded antenna pulling in workers of two species of formicine ants, *Formica cinerea* and *Formica fusca*, in the context of rescue of a victim attacked by an antlion larva. Interestingly, the single case of rescue behaviour displayed in response to a homospecific nestmate reported by these authors in *Formica fusca* consisted of antenna pulling.

Antenna pulling was also mentioned in the lists of behavioural categories used in two further studies of rescue behaviour of workers of *Formica cinerea* and in a comparative study of rescue behaviour of workers of *Formica cinerea* and five other ant species from Borneo and Poland [60,61,62]. However, the description of the results of these last three studies does not contain any information if antenna pulling has actually been observed in these experiments.

Crawling under the piece of filter paper used as a part of the snare apparatus that has been observed in workers of *Cataglyphis piliscapa* after futile attempts to bite through the snare [19] took place in our experiment, too, but was very infrequent and only in one case followed unsuccessful rescue attempts directed towards the wire loop on the victim’s petiole. Therefore, on the basis of our findings this behaviour cannot be considered to act as a rescue tactic to which *Formica polyctena* workers may turn after unsuccessful attempts to break the snare.

### 4.7. Importance of the Results of this Study and the Need of Further Comparative Research

Our present experiment demonstrated that workers of the red wood ant *Formica polyctena* did not direct their rescue attempts preferentially towards the snare responsible for the immobilization of a nestmate victim, but directed them indiscriminately towards the wire loops implicated and not implicated in restraining the victim’s movements. This finding throws an important light not only on cognitive processes involved in the mediation of ant rescue behaviour, but also on cognitive abilities of invertebrates in general. 

However, the conclusions drawn on the basis of these findings can be applied legitimately only to workers of the tested species, *Formica polyctena* and should not be extended automatically to other ant species without further comparative research. Such prudence has to be recommended, as comparative research on ant rescue behaviour revealed a very large number of important interspecific differences. To name just a few of them, rescue behaviour was found to be fully expressed only in some ant species, whereas in other species it proved to be absent or limited to very short episodes [59,61,67]. Some ants were also found to engage in rescue behaviour only in some specific contexts. For instance, workers from monospecific colonies of *Formica fusca* (subfamily Formicinae) did not rescue nestmates captured by antlion larvae [28], but readily rescued nestmate victims during artificial snare bioassays [69]. The same proved to be true for workers of the dolichoderine ant species *Iridomyrmex anceps* [61]. Similarly, shortened life expectancy influenced rescue behaviour of workers of *Formica cinerea* during artificial snare bioassays [67,80], but not during antlion larva capture bioassays [62].

Other interspecific differences in ant rescue behaviour included presence/absence of antenna pulling (already discussed in the Section 4.6) and presence/absence of digging behaviour during rescue actions performed by the ants to bring help to victims of antlion larvae. While some ants (in particular *Formica cinerea*) were repeatedly reported to engage in digging behaviour during their attempts to rescue victims of antlion larvae [28,60,61,62,63], such behaviour was never performed by workers of a myrmicine ant species *Tetramorium* sp. E [59]. Absence of that subcategory of rescue behaviour was even interpreted as an illustration of advanced cognitive abilities of these ants, as the authors of that study argued that digging could cause the collapse of the walls of the antlion pit and exacerbate the problem [59].

Ants from various species were also found to differ with respect to the propensity to rescue non-nestmates. Thus, *Cataglyphis piliscapa*, *Cataglyphis floricola* and *Lasius grandis* workers did not rescue mature non-nestmate conspecific workers entrapped in artificial snares [29,67]. However, in a study with the use of the same bioassay in which potential rescuers of *Cataglyphis piliscapa* had to respond to very young victims (the so called callows), rescue behaviour was triggered not only by nestmate and non-nestmate conspecifics, but also by allospecifics (*Camponotus aethiops*) [65]. Expression of rescue behaviour in response to mature conspecific non-nestmate workers was also documented in *Tetramorium* sp. E (subfamily Myrmicinae) [59], *Oecophylla smaragdina* (subfamily Formicinae) [71] and *Odontomachus brunneus* (subfamily Ponerinae) [73].

Interspecific differences were also detected by the studies carried out to investigate rescue behaviour directed towards various subcategories of nestmate victims. Thus, ants from the species *Megaponera analis* were found to respond differently to lightly and heavily injured nestmates and to living and dead ones [47,48]. Injured workers of *Formica cinerea* (but not intact ones) also discriminated between intact and injured nestmates and engaged in less intense rescue attempts during their confrontations with the latter [75]. However, workers of *Cataglyphis nigra* responded similarly to intact and injured victims and even to living and dead ones [77].

Finally, secretions of mandibular glands were found to be implicated in chemical signalling adopted by the victims to summon the rescuers in the case of ant species *Megaponera analis* (subfamily Ponerinae) [95] and *Veromessor pergandei* (subfamily Myrmicinae) [72], but not in the case of *Formica cinerea* (subfamily Formicinae) [70].

All these examples of profound interspecific differences documented by the studies on various aspects of ant rescue behaviour highlight the importance of comparative research for reliability of general conclusions drawn on the basis of experimental findings obtained for specific ant species tested in specific contexts.

### 4.8. Perspectives of Future Research

Our present study opens interesting perspectives of comparative research involving the use of our new bioassay: artificial snare bioassay with two wire loops, one placed on the victim’s petiole and acting as a snare and an additional one on the victim’s leg. Such research involving more colonies of *Formica polyctena* and more ant species is indispensable if we want to learn how general the validity of the conclusions of our present study is.

We also would like to point out that we have still to report an important part of findings of our present experiment, including in particular the data on behaviour patterns not classified as subcategories of rescue behaviour. That future publication will also include the results of comparisons of behaviour of not only various subgroups of rescuers, but also of rescuers versus non-rescuers.

### 4.9. Conclusions

Our present study sheds an interesting new light on cognitive processes underlying rescue behaviour directed by ants to their immobilized nestmates and, in particular, on the question of the ability of these insects to identify precisely the source of the victim’s restraint. As revealed by our present findings, the overall strategy adopted by the most active rescuers of the red wood ant *Formica polyctena* does not consist solely or even only preferentially of rescue attempts precisely targeted to the source of the victim’s entrapment, the snare on its petiole. Instead, it consists of intense versatile switching between various subcategories of rescue behaviour. 

However, this conclusion cannot be automatically extended to all possible contexts and all ant species, as ant rescue behaviour is strongly context-dependent and shows many striking interspecific differences. Only further comparative research will allow us to find out how general is the validity of our present conclusions.

As we documented many important differences between the behaviour of various subclasses of rescuers investigated in our experiment, our study also contributed to broadening of our knowledge concerning the diversity and variability of specific patterns of ant behaviour. We also introduced a new version of the artificial snare bioassay that may be used in the future experimental research aiming at better understanding of factors involved in the mediation of rescue behaviour of ants and other animals. 

Lastly, our present findings also highlight the importance of rigorous experimental testing of precisely formulated hypotheses for more profound understanding of causal factors underlying cognitive abilities of animals.

## Figures and Tables

**Table 1:** Operational definitions of behavioural categories used to quantify rescue behaviour shown by individually marked workers of the red wood ant *Formica polyctena* during nestmate rescue tests.

Behavioural Category	Operational Definition
Rescue attempts directed towards various parts of the victim’s body	
Pulling the victim’s leg	The ant uses its mandibles to grab the victim’s leg and to pull it backwards
Pulling the victim’s neck	The ant uses its mandibles to grab the victim’s neck and to pull it backwards
Pulling the victim’s mandible	The ant uses its mandibles to grab the victim’s mandible and to pull it backwards
Pulling the victim’s antenna ^1^	The ant uses its mandibles to grab the victim’s antenna and to pull it backwards
Pulling or levering of another part of the victim’s body	The ant uses its mandibles to grab the victim’s thorax or abdomen and/or to pull/lever the victim’s body
Rescue attempts directed towards the substrate near the victim ^2^	
Sand digging	The ant engages in sand digging near the victim with the use of its legs and sometimes also its mandibles
Sand transport	The ant uses its mandibles to transport small pebbles away from the victim
Pulling the paper disc	The ant uses its mandibles to pull the paper disc to which the victim is tied
Crawling under the paper disc	The ant is active under the paper disc, but is concealed from the view of the camcorder
Rescue attempts directed towards the wire loop on the victim’s petiole	
Biting and/or pulling the wire loop on the victim’s petiole	The ant uses its mandibles to grab the wire loop on the victim’s petiole and to bite and/or pull it (behaviour engaging the whole body of the rescuer)
Nibbling at the wire loop on the victim’s petiole	The ant uses its mandibles to grab the wire loop on the victim’s petiole and then engages in its nibbling (prudent repeated movements of mandibles)
Biting/pulling behaviour directed towards the wire loop on the victim’s petiole or very close to it (within ±1 mm)	The ant engages in biting/pulling behaviour directed towards the wire loop on the victim’s petiole or to its close vicinity, but is bent over the victim and its mandibles are hidden from the view of the camcorder
Rescue attempts directed towards the wire loop on the victim’s leg	
Biting and/or pulling the wire loop on the victim’s leg	The ant uses its mandibles to grab the wire loop on the victim’s leg and to bite and/or pull it (behaviour engaging the whole body of the rescuer)
Nibbling at the wire loop on the victim’s leg	The ant uses its mandibles to grab the wire loop on the victim’s leg and then engages in its nibbling (prudent repeated movements of mandibles)
Biting/pulling behaviour directed towards the wire loop on the victim’s leg or very close to it (within ±1 mm)	The ant engages in biting/pulling behaviour directed towards the wire loop on the victim’s leg or to its close vicinity, but is bent over the victim and its mandibles are hidden from the view of the camcorder

^1^ This category (pulling the victim’s antenna) was never observed during the whole experiment. ^2^ The expression “near the victim” means that the behaviour in question took place at a distance of less than one body length of a worker of *Formica polyctena*.

**Table 2:** Comparison of responses of workers of the red wood ant *Formica polyctena* (WL+ ants) to wire loops placed on the leg and on the petiole of a nestmate victim entrapped in an artificial snare. (A) All responses of WL+ ants to the wire loop on the leg (L) and on the petiole (P) of the victim (*n* = 36 ants). (B) Responses of WL+ ants to the wire loop on the immobile leg (LI) and on the petiole (P) of the victim (*n* = 34 ants *). WL+ ants: ants that engaged in rescue behaviour including the responses to one or both wire loops placed on the victim’s body. Latency [%]: the latency from the start of the test to the start of the first episode of biting/pulling of the wire loop expressed as the percentage of the total test time. Number of episodes: the total number of episodes of biting/pulling of the wire loop recorded during the test (values reported with the accuracy of ±1). Total duration [s]: the total duration of all episodes of biting/pulling of the wire loop recorded during the test. Test duration: 20 min. Statistics: Wilcoxon matched-pairs signed-rank test.

A. All responses of WL+ ants to the wire loops on the leg (L) and the petiole (P) of the victim (*n* = 36 ants)					
Variable	Measure	Leg (L)	Petiole (P)	*Z*	*p*
Latency [%]	Median	59.17	64.66	−0.20	0.838 (NS)
	Quartiles	24.38–100.00	17.17–100.00		
	Range	3.44–100.00	3.32–100.00		
Number of episodes	Median	1	1	−1.02	0.309 (NS)
	Quartiles	0–5	0–3		
	Range	0–17	0–14		
Total duration [s]	Median	7.76	3.34	−1.46	0.144 (NS)
	Quartiles	0–34.21	0–21.89		
	Range	0–171.00	0–331.24		
B. Responses of WL+ ants to the wire loops on the immobile leg (LI) and the petiole (P) of the victim (*n* = 34 ants) *					
Variable	Measure	Leg (LI) (immobile)	Petiole (P)	*Z*	*p*
Latency [%]	Median	67.02	60.07	−0.56	0.578 (NS)
	Quartiles	24.61–100.00	16.29–100.00		
	Range	3.44–100.00	3.32–100.00		
Number of episodes	Median	1	1	−0.53	0.595 (NS)
	Quartiles	0–5	0–3		
	Range	0–17	0–14		
Total duration [s]	Median	10.50	3.48	−1.24	0.215 (NS)
	Quartiles	0–37.77	0–25.48		
	Range	0–171.00	0–331.24		

* Two L ants that responded to the wire loop on the victim’s leg only when that leg was in movement have been discarded from the analysis (B).

**Table 3:** Responses of workers of the red wood ant *Formica polyctena* to the wire loop on the victim’s leg carried out while that leg was in movement (LM). Only six ants that were observed to engage in that behaviour have been taken into account. Each ant is identified by the number of the test in which it participated and the first letter of the name of its colour mark (b: blue, g: green, v: violet, y: yellow). L: ants that directed their rescue attempts only to the wire loop on the victim’s leg (L). L+P: ants that directed their rescue attempts to both wire loops. RL: Total number of responses to the wire loop on the victim’s leg. RLM: Total number of responses to the wire loop on the victim’s leg taking place when that leg was in movement. B: rescue attempts directed towards the victim’s body. P: rescue attempts directed towards the wire loop on the victim’s petiole. Test duration: 20 min.

Ant	Subcategory	RL	RLM	First Episode of Rescue Behaviour
5b	L	1	1	B
17v	L	2	1 *	B
18v	L	1	1	B
4g	L+P	7	1	B
6y	L+P	17	3 **	P
14g	L+P	1	1	P

* Response performed as the 2nd bout of rescue behaviour directed towards the loop on the victim’s leg. ** Responses performed as the 4th, 7th and 8th bout of rescue behaviour directed towards the loop on the victim’s leg.

**Table 4:** Comparison of the length [in mm] of the wire loops placed on the leg (L) and the petiole (P) of workers of the red wood ant *Formica polyctena* (*n* = 30) used as victims in the nestmate rescue tests with the use of the artificial snare. In the case of the loop on the petiole (P) only the part of the wire accessible for the potential rescuer ants (not hidden under the paper disc to which the victim was tied) was taken into account in the measurements. Statistics: Wilcoxon matched-pairs signed-rank test.

Variable	Measure	Leg (L)	Petiole (P)	*Z*	*p*
Length of the accessible part of the wire loop [mm]	Median	3.3	3.5	−1.31	0.191 (NS)
	Quartiles	2.6–3.7	3.0–3.8		
	Range	2.2–5.5	2.0–5.2		

## Data Availability

Additional data can be found in keeping of the Laboratory of Ethology of the Nencki Institute of Experimental Biology PAS (Warsaw, Poland) and they are accessible upon reasonable request from the corresponding author.

## Supplementary Materials

The following supporting information can be downloaded at: https://www.mdpi.com/article/10.3390/life14040515/s1. Table S1: Total duration [s] of rescue behaviour displayed by individual workers of the red wood ant *Formica polyctena* (WL– ants, *n* = 17) in response to a nestmate victim entrapped in an artificial snare; Table S2: Successive subcategories of rescue behaviour displayed by individual workers of the red wood ant *Formica polyctena* (WL– ants, *n* = 17) in response to a nestmate victim entrapped in an artificial snare; Table S3: Total duration [s] of rescue behaviour and of its two main subcategories displayed by individual workers of the red wood ant *Formica polyctena* (L ants, *n* = 12) in response to a nestmate victim entrapped in an artificial snare; Table S4: Successive subcategories of rescue behaviour displayed by individual workers of the red wood ant *Formica polyctena* (L ants, *n* = 12) in response to a nestmate victim entrapped in an artificial snare; Table S5: Total duration [s] of rescue behaviour and of its three main subcategories displayed by individual workers of the red wood ant *Formica polyctena* (L+P ants, *n* = 12) in response to a nestmate victim entrapped in an artificial snare; Table S6: Successive subcategories of rescue behaviour displayed by individual workers of the red wood ant *Formica polyctena* (L+P ants, *n* = 12) in response to a nestmate victim entrapped in an artificial snare; Table S7: Total duration [s] of rescue behaviour and of its two main subcategories displayed by individual workers of the red wood ant *Formica polyctena* (P ants, *n* = 12) in response to a nestmate victim entrapped in an artificial snare; Table S8: Successive subcategories of rescue behaviour displayed by individual workers of the red wood ant *Formica polyctena* (P ants, *n* = 12) in response to a nestmate victim entrapped in an artificial snare; Video S1: Rescue behaviour of *Formica polyctena* 1; Video S2: Rescue behaviour of *Formica polyctena* 2; Video S3: Rescue behaviour of *Formica polyctena* 3; Video S4: Rescue behaviour of *Formica polyctena* 4; Video S5: Rescue behaviour of *Formica polyctena* 5; PDF File S1: Contents of short videos showing rescue behaviour of *Formica polyctena* workers.
