# Supplementary material for: Do They Know What They Are Doing? Cognitive Aspects of Rescue Behaviour Directed by Workers of the Red Wood Ant Formica polyctena to Nestmate Victims Entrapped in Artificial Snares
Source: Life (Basel). 2024 Apr 16;14(4):515. doi: 10.3390/life14040515 (PMC11051173; doi:10.3390/life14040515)
Supplement: Supplementary file 1 [file life-14-00515-s001.zip › Contents of short videos showing rescue behaviour of workers of Formica polyctena.pdf]

Contents of videos illustrating rescue behaviour of individually marked workers of the red wood ant *Formica polyctena* recorded during nestmate rescue tests consisting of a confrontation with a nestmate victim bearing on its body two wire loops: a loop on the petiole acting as a snare and a loop on the leg not implicated in the victim's entrapment

The names of colours given in the description of the content of movies denote the colours of paint used to mark the tested workers of *Formica polyctena.*

(1) Rescue Behaviour of *Formica polyctena* 1.wmv (1'59")

Blue ant: sand transport, sand digging, biting/pulling of the wire loop on the victim's petiole, pulling of the victim's leg not bearing a wire loop, sand digging.
Yellow ant: very short episodes of sand digging, biting/pulling of the wire loop on the victim's leg.

(2) Rescue Behaviour of *Formica polyctena* 2.wmv (1'29")

Yellow ant: persistent biting/pulling of the wire loop on the victim's leg.
Red ant: attempts at levering of the victim's abdomen, sand digging, pulling of the victim's leg not bearing a wire loop.

(3) Rescue Behaviour of *Formica polyctena* 3.wmv (1'00")

Yellow ant: persistent biting/pulling of the wire loop on the victim's petiole.

(4) Rescue Behaviour of *Formica polyctena* 4.wmv (1'00")

Green ant: sand digging, persistent biting/pulling of the wire loop on the victim's leg.
Yellow ant: pulling of the victim's leg not bearing a wire loop.

(5) Rescue Behaviour of *Formica polyctena* 5.wmv (43")

Yellow ant: short sand digging, persistent biting/pulling of the wire loop on the victim's petiole and its vicinity.
Red ant: pulling of the victim's leg not bearing a wire loop.
