# Supplementary material for: Do They Know What They Are Doing? Cognitive Aspects of Rescue Behaviour Directed by Workers of the Red Wood Ant Formica polyctena to Nestmate Victims Entrapped in Artificial Snares
Source: Life (Basel). 2024 Apr 16;14(4):515. doi: 10.3390/life14040515 (PMC11051173; doi:10.3390/life14040515)
Supplement: Supplementary file 1 [file life-14-00515-s001.zip › Szczuka et al. Supplementary online materials Tables S1-S8.pdf]

Article

Do They Know What They Are Doing? Cognitive Aspects of Rescue Behaviour Directed by Workers of the Red Wood Ant *Formica polyctena* to Nestmate Victims Entrapped in Artificial Snares

Anna Szczuka [1], Alicja Sochacka-Marlowe [1,2], Julita Korczyńska [1], Paweł Jarosław Mazurkiewicz [1,3], Beata Symonowicz [1], Olga Kukina [1,4] and Ewa Joanna Godzińska [1,*]

[1] Laboratory of Ethology, Nencki Institute of Experimental Biology of the Polish Academy of Sciences, Ludwika Pasteura St. 3, PL 02-093 Warsaw, Poland; a.szczuka@nencki.edu.pl (A.S.); alamarlowe@gmail.com (A.S.-M.); j.korczynska@nencki.edu.pl (J.K.); pawel.j.mazurkiewicz@gmail.com (P.J.M.); beata_sym@o2.pl (B.S.); ol.kukina@gmail.com (O.K.)

[2] Department of Biology and Integrated Bioscience Program, University of Akron, Akron, OH 44325, USA

[3] College of Inter-Faculty Individual Studies in Mathematics and Natural Sciences (MISMaP), University of Warsaw, Stefana Banacha St. 2c, PL 02-097 Warsaw, Poland

[4] Department of Entomology, Phytopathology and Physiology, Ukrainian Research Institute of Forestry and Forest Melioration, Pushkinska St. 86, 61024 Kharkiv, Ukraine

* Correspondence: e.godzinska@nencki.edu.pl

Table S1. Total duration [s] of rescue behaviour displayed by individual workers of the red wood ant *Formica polyctena* (WL- ants, *n* = 17) in response to a nestmate victim entrapped in an artificial snare. WL- ants: ants that engaged in rescue behaviour directed to the victim's body (b) or to the substrate in its vicinity (s), but never directed it to any of the wire loops (WL) placed on the victim's body. The values of the total duration of rescue behaviour are presented in ascending order. Each ant is identified by the number of the test in which it participated and the first letter of the name of its colour mark (b: blue, g: green, r: red, v: violet, y: yellow).

Ant	Total duration [s] of rescue behaviour [rescue attempts directed to the victim's body (b) and to the substrate (s) near the victim]
11g	0.08
10g	0.72
6g	1.36
11y	1.76
3r	2.84
2v	3.48
14y	3.84
6v	5.80
16g	6.72
6r	7.28
10r	7.52
7b	9.00
6b	17.12
9v	20.76
7g	28.32
8b	88.88
24v	153.88

Table S2. Successive subcategories of rescue behaviour displayed by individual workers of the red wood ant *Formica polyctena* (WL- ants, *n* = 17) in response to a nestmate victim entrapped in an artificial snare. The sequences of bouts of two main types of rescue attempts (1-8 elements; number of elements of each sequence is indicated in parentheses) are presented in ascending order. If successive bouts of the same subcategory of rescue behaviour were not separated by behaviour belonging to another subcategory of rescue attempts, they are represented as a single element of the sequence even if they were interspersed by bouts of behaviour not consisting of rescue attempts. Other explanations as in Table S1.

Ant	Successive subcategories of rescue behaviour (b *vs* s)
3r	s (1)
6b	s (1)
6g	s (1)
6r	s (1)
6v	s (1)
7b	s (1)
7g	s (1)
9v	s (1)
10r	s (1)
11g	s (1)
11y	s (1)
10g	b (1)
14y	b (1)
16g	b (1)
2v	b (1)
8b	bsbsbsb(7)
24v	bsbsbsbs (8)

Table S3. Total duration [s] of rescue behaviour and of its two main subcategories displayed by individual workers of the red wood ant *Formica polyctena* (L ants, *n* = 12) in response to a nestmate victim entrapped in an artificial snare. L ants: ants that at least once directed their rescue attempts to the wire loop on the victim's leg (L), but never directed them to the wire loop on the victim's petiole. b, s, L: rescue attempts directed, respectively, to the victim's body (b), to the substrate near the victim (s), and to the wire loop on its leg (L). The values of the total duration of all subcategories of rescue behaviour pooled together are presented in ascending order. Other explanations as in Table S1.

Ant	Total duration [s] of all subcategories of rescue behaviour pooled together	Total duration [s] of rescue behaviour directed to the victim's body or to the substrate near the victim (b+s)	Total duration [s] of rescue behaviour directed to the wire loop on the victim's leg (L)
25v	5.60	0	5.60
18v	6.24	3.80	2.44
19v	12.55	1.47	11.08
13v	22.12	20.48	1.64
1r	40.80	24.08	16.72
5b	73.20	70.28	2.92
17v	157.49	144.69	12.80
21b	188.72	157.84	30.88
3g	359.44	327.72	31.72
4y	453.04	383.96	69.08
10v	659.58	521.58	138.00
19r	1097.58	926.58	171.00

Table S4. Successive subcategories of rescue behaviour displayed by individual workers of the red wood ant *Figure 12.* in response to a nestmate victim entrapped in an artificial snare. The sequences of bouts of various types of rescue attempts (1-75 elements; number of elements of each sequence is indicated in parentheses) are presented in ascending order. Other explanations as in Tables S2 and S3.

Ant	Successive subcategories of rescue behaviour
25v	L (1)
3g	Lb (2)
19v	Ls (2)
18v	bL (2)
1r	bLbsb (5)
5b	bLsbs (5)
13v	sbLsbs (6)
17v	bsbLsbsL (8)
21b	bsbLbsbsbsLsbsb (15)
4y	bLbLbLbLbLbLbsbLbsbs (20)
10v	bsbsbsbsbsbsbsbsLsLsbsLsLsbLsLbsbsbsbsbsbsbsbsbs (51)
19r	sbLsbsbLbsLbsbsbsbsLsLsLsLsbsbLbsbsLbLsLbsLsbLsbsbLsbsLbsbsbsbsbsbsLsbsbL (75)

Table S5. Total duration [s] of rescue behaviour and of its three main subcategories displayed by individual workers of the red wood ant *Formica polyctena* (L+P ants, n = 12) in response to a nestmate victim entrapped in an artificial snare. L+P ants: ants that directed their rescue attempts to both wire loops placed on the victim's body, the one on its leg (L) and the one on its petiole (P). b+s, L, P, L+P: rescue attempts directed, respectively, to the victim's body (b) and the substrate near the victim (s), to the wire loop on the victim's leg (L), to the wire loop on the victim's petiole (P), and to both wire loops on the victim's body. The values of the total duration of all subcategories of rescue behaviour pooled together are presented in ascending order. Other explanations as in Table S1.

Ant	Total duration [s] of all subcategories of rescue behaviour pooled together	Total duration [s] of rescue behaviour directed to the victim's body and to the substrate near it (b+s)	Total duration [s] of rescue behaviour directed to the wire loop on the victim's leg (L)	Total duration [s] of rescue behaviour directed to the wire loop on the victim's petiole (P)	Total duration [s] of rescue behaviour directed to both wire loops on the victim's body (L+P)
14g	39.60	27.52	3.28	8.80	12.08
9b	212.28	184.76	24.28	3.24	27.52
22b	344.84	212.08	124.96	7.80	132,76
13r	345.04	325.32	13.60	6.12	19.72
13y	432.62	237.26	128.28	7.08	195.3
6y	445.34	337.42	65.64	42.28	107.92
7y	451.28	408.72	3.40	39.16	42.56
12y	455.72	392.44	35.04	28.24	63.28
11v	625.27	538.11	58.24	28.92	87.16
4g	665.82	610.14	48.16	7.52	55.68
3y	731.52	369.28	31.00	331.24	362.24
10b	806.93	767.97	14.40	24.56	38.96

Table S6. Successive subcategories of rescue behaviour displayed by individual workers of the red wood ant *Figure 12.* in response to a nestmate victim entrapped in an artificial snare. b, s, L, P: rescue attempts directed, respectively, to the victim's body (b), to the substrate in its vicinity (s), to the wire loop on its leg (L), and to the wire loop on its petiole (P). The sequences of bouts of various types of rescue attempts (6-72 elements; number of elements of each sequence is indicated in parentheses) are presented in ascending order. Other explanations as in Tables S2 and S5.

Ant	Successive subcategories of rescue behaviour
14g	PbPbLb (6)
9b	bLbsbLbLbsbsbP (14)
4g	bLbPLb LbL bLb LbL (15)
22b	bLbLbLbPbLbLbsbsb (17)
3y	sbsbsbsLbsbsLbsPsbLbPb (22)
13r	sbsbPsbsbsbsbsbsLbsbsbsbsLsbsbsbsbsbPb (38)
7y	bPbsbsbsbsbsbsbsbsbPbsPsbsPLPbsbsbsbs (39)
10b	sPbsbPsbsbsbsbPbPbPbsbsbsbsbLbsbsbsbPbsbsb (42)
12y	sLbsbsLsbsbsbsbsbsbsbsbsPsPsbsLbLsbsbsbsLbs (43)
13y	bsbPbsPsbsPbPLbsbPsbPbsbsbsbLsLbsbsbsLsbsbsbsbL (47)
11v	sbPbLPsLbPbsbLsbsbsbsbsbsbsbsbsLPsLsPbsLsbsbPsPbsbsLsLsbs bsLbsbsbsbsbsb (71)
6y	PbPbsbLPbLbsbLbPbPsLbPbsbsbLbPbLbLbPbsbsbLbLbPbLbLbPbP bLbPbLbLbPLbPbsbLb (72)

Table S7. Total duration [s] of rescue behaviour and of its two main subcategories displayed by individual workers of the red wood ant *Formica polyctena* (P ants, *n* = 12) in response to a nestmate victim entrapped in an artificial snare. P ants: ants that at least once directed their rescue attempts to the wire loop on the victim's petiole (P), but never directed them to the wire loop on the victim's leg. b, s, P: rescue attempts directed, respectively, to the victim's body (b), to the substrate near the victim (s), and to the wire loop on its petiole (P). The values of the total duration of all subcategories of rescue behaviour pooled together are presented in ascending order. Other explanations as in Table S1.

Ant	Total duration [s] of all subcategories of rescue behaviour pooled together	Total duration [s] of rescue behaviour directed to the victim's body or to the substrate near the victim (b+s)	Total duration [s] of rescue behaviour directed to the wire loop on the victim's petiole (P)
15v	7.36	3.80	3.56
20v	10.08	6.68	3.40
12v	22.80	21.56	1.24
10y	32.84	29.56	3.28
1v	44.72	42.32	2.40
15g	73.80	59.92	13.88
17r	82.16	81.12	1.04
20g	138.56	126.52	12.04
23b	144.32	142.12	2.20
1b	182.12	132.92	49.20
12g	198.16	193.76	4.40
2r	753.72	639.32	114.40

Table S8. Successive subcategories of rescue behaviour displayed by individual workers of the red wood ant *Figure 12.* in response to a nestmate victim entrapped in an artificial snare. The sequences of bouts of various types of rescue attempts (2-45 elements; number of elements of each sequence is indicated in parentheses) are presented in ascending order. Other explanations as in Tables S2 and S7.

Ant	Successive subcategories of rescue behaviour
15v	bP (2)
20v	bP (2)
12v	sbPs (4)
17r	bPbsb (5)
1v	bsbPb (5)
15g	PbPbPb (6)
10y	bsbsbPb (7)
20g	bPbPbPsbs (8)
1b	bPbsbsbsPbsbsbsbsbPbPsP (23)
12g	bsbsbsbsbPsbsbsPsbsbsbsbs (25)
23b	sbsPsbsbsbsbsbsbsbsbsbsbsb (26)
2r	bsbsbsbsbsbsbsbsbsbsbsbsbsbsbsbsbPbPbPbPsbPsb (45)

Disclaimer/Publisher's Note: The statements, opinions and data contained in all publications are solely those of the individual author(s) and contributor(s) and not of MDPI and/or the editor(s). MDPI and/or the editor(s) disclaim responsibility for any injury to people or property resulting from any ideas, methods, instructions or products referred to in the content.
